# Flexible modeling of regulatory networks improves transcription factor activity estimation

**DOI:** 10.1038/s41540-024-00386-w

**Published:** 2024-05-28

**Authors:** Chen Chen, Megha Padi

**Affiliations:** 1 Department of Epidemiology and Biostatistics, University of Arizona Mel and Enid Zuckerman College of Public Health, Tucson, AZ USA; 2 University of Arizona Cancer Center, University of Arizona, Tucson, AZ USA; 3 Department of Molecular and Cellular Biology, University of Arizona, Tucson, AZ USA

**Keywords:** Software, Cancer

## Abstract

Transcriptional regulation plays a crucial role in determining cell fate and disease, yet inferring the key regulators from gene expression data remains a significant challenge. Existing methods for estimating transcription factor (TF) activity often rely on static TF-gene interaction databases and cannot adapt to changes in regulatory mechanisms across different cell types and disease conditions. Here, we present a new algorithm - Transcriptional Inference using Gene Expression and Regulatory data (TIGER) - that overcomes these limitations by flexibly modeling activation and inhibition events, up-weighting essential edges, shrinking irrelevant edges towards zero through a sparse Bayesian prior, and simultaneously estimating both TF activity levels and changes in the underlying regulatory network. When applied to yeast and cancer TF knock-out datasets, TIGER outperforms comparable methods in terms of prediction accuracy. Moreover, our application of TIGER to tissue- and cell-type-specific RNA-seq data demonstrates its ability to uncover differences in regulatory mechanisms. Collectively, our findings highlight the utility of modeling context-specific regulation when inferring transcription factor activities.

## Introduction

A critical aspect of systems biology is to understand transcriptional regulation, i.e., to identify regulators driving a particular function and to determine the activities of those transcription factors (TFs)^1^. However, the challenge is that TF activity is difficult to measure, and the mRNA expression of a TF is not a reliable surrogate for its activity. Furthermore, the targets of these TFs can change depending on the cell type, disease context, epigenetic state, and many other factors. Because of these problems, many computational techniques have emerged to infer regulatory networks and the associated TF activity (TFA) levels.

One class of algorithms for inferring TFA from transcriptomic data uses prior knowledge about the target genes of the TF. For instance, VIPER determines TFA by searching for differential expression in the target genes (or “regulon”) of each transcription factor, while taking into account the mode of the regulatory interactions (activation or inhibition)^2^. Regulons can be defined using consensus interactions from a public database or by applying a network inference algorithm to identify context-specific target genes. Studies have demonstrated that, for both bulk RNA-seq and single-cell RNA-seq data, VIPER achieves better results when using high-confidence consensus regulons from curated databases, rather than context-specific regulons inferred from data using network inference methods like ARACNe^3,4^. Consensus regulon databases like DoRothEA^3^ are also recommended for use with many other TFA inference methods, including GSEA (Gene Set Enrichment Analysis) ULM (Univariate Linear Regression)^5^ and others implemented in the R package decoupleR. This suggests that the advantage of including computationally inferred regulatory interactions is offset by the lower accuracy of those interactions.

Optimally, prior knowledge about consensus TF regulons should be updated using expression data to jointly learn context-specific interactions and TF activity levels. Several methods have explored this approach. The Inferelator^6^ uses a two-step process where linear regression is first used to infer TFA from gene expression data and TF binding priors, and then network edge weights are updated using regularized regression. Similar to VIPER, the TFA calculation here depends only on known regulatory interactions and is independent of the context-specific networks learned from the expression data. Some other methods simultaneously learn networks and TF activity levels but are limited in their ability to flexibly incorporate prior knowledge on mode of regulation. Network Component Analysis (NCA) introduced the concept of using matrix factorization to infer latent TFA from static prior networks^7^. Recent advancements integrate network inference with probabilistic models for a more refined estimate of TF activity, but they estimate TF-TG binding using mutual information without incorporating prior knowledge of TF binding^8^. The constrained matrix factorization (CMF) method from^9^ jointly estimates TFA and the regulatory network, but all the regulatory interactions are fully constrained to be activating or inhibiting and cannot adapt to the input data. BITFAM^10^ was developed to estimate regulatory networks and TFA using scRNA-seq data but does not consider whether interactions are activating or inhibiting. Techniques leveraging time series gene expression data excel in modeling regulatory elements like feed-forward loops and autoregulation, enhancing TF activity estimation, but are limited by availability of time series data^11^. Some methods provide improved activity estimation for TFs which have been profiled by ChIP-seq in the cell type of interest, but these are limited to a small subset of TFs that have such information available^12^. Our focus is on improving TF activity and gene regulatory network estimation using static gene expression data and widely available prior knowledge, aiming to fill the gap where direct interactions and time series data are unavailable.

Here, we introduce a new method called TIGER (Transcriptional Inference using Gene Expression and Regulatory data) that jointly infers a context-specific regulatory network and corresponding TF activity levels while adaptively incorporating information on consensus target genes and their mode of regulation. TIGER sets itself apart from other approaches by its ability to integrate and adjust pre-existing knowledge about the mode and strength of regulation. This is made possible through TIGER’s implementation of a Bayesian framework, which can judiciously incorporate network sparsity and edge sign constraints by applying tailored prior distributions to the variables. In addition, we implement a Variational Bayesian method^13,14^ to accelerate the estimation process. When applied to yeast, cancer cell lines, normal breast tissue, and cell-type-specific RNA-seq data, TIGER can more sensitively infer condition-specific TF activities than comparable methods. The improved accuracy of TIGER demonstrates that integrating both high-confidence regulon databases and context-specific regulatory features in a principled manner can better identify master regulators and disease mechanisms.

## Results

### Overview of TIGER

**Fig. 1:**
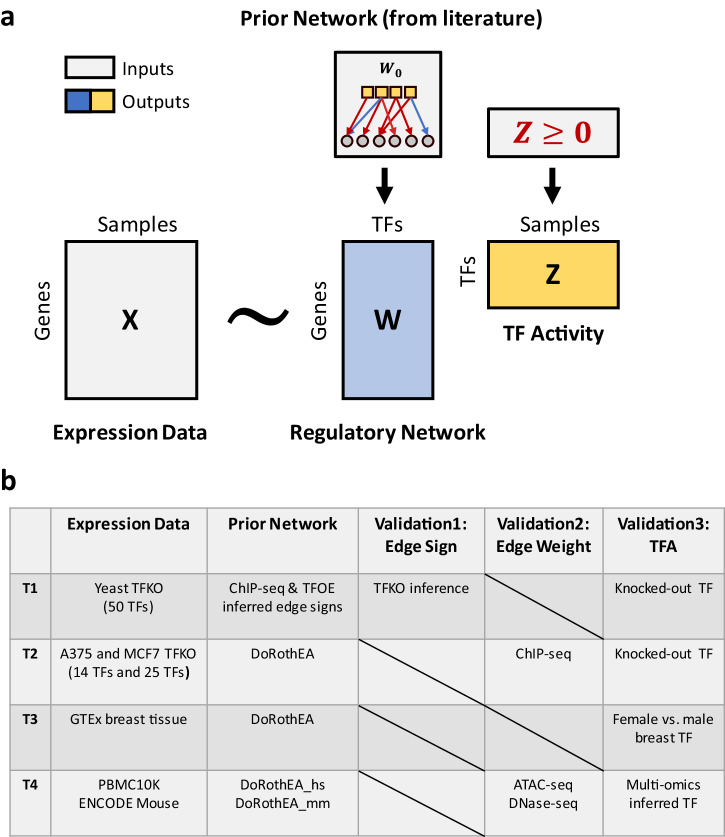
Overview of TIGER. **a** TIGER uses a matrix decomposition method to estimate regulatory network $W$ and TF activity $Z$. The inputs of TIGER are normalized gene expression matrix $X$ and prior TF binding network ${W}_{0}$ curated from the literature. Prior binding information is incorporated as different prior distributions in matrix $W$. Matrix $Z$ is constrained to be strictly non-negative for identifiability and biological interpretation. Variational Inference is used to estimate parameters. **b** Description of the validation strategy. Test 1 (T1) uses yeast TF Knockout (TFKO), TF Overexpression (TFOE), and ChIP-seq data to assess the impact of sign-flipping. Test 2 (T2) uses A375 and MCF7 cancer cell line TFKO datasets and the DoRothEA prior network to assess the importance of edge weights. Tests 3 and 4 (T3 and T4) explore TIGER’s potential in tissue-specific and cell-type-specific data analysis.

TIGER estimates a gene regulatory network (GRN) and TF activity (TFA) levels through a matrix factorization framework (Fig. 1a). Specifically, we aim to decompose an observed log-transformed normalized gene expression matrix $X$ into a product of two matrices, $W$ and $Z$, where $W$ is interpreted as a regulatory network (with rows corresponding to genes and columns corresponding to TFs), and $Z$ is interpreted as a TFA matrix (with TFs as rows and samples as columns). To guide the construction of biologically meaningful GRN and TFA, we impose both network structure and edge sign constraints to $W,$ and constrain the TFA to be strictly non-negative. The non-negative constraint on the TFA matrix breaks the symmetry in the edge signs in the GRN matrix and guarantees that they can be interpreted as activation and suppression events. As recommended in decoupleR and other publications, we use a high-confidence consensus database like DoRothEA to formulate a prior GRN. We use a Bayesian framework to update the prior knowledge and constraints using the gene expression data. Since the edges in a consensus database may be overly generic and not all active in the same condition, we employ a sparse prior to filter out context-irrelevant edges. Additionally, since the mode of regulation is often unknown or erroneous, we have implemented a prefiltering step to constrain only those edge signs that are consistently annotated among two independent sources of prior knowledge. Other edges remain unconstrained in their mode of regulation, and their sign is learned directly from the data. Please see the Methods and Supplementary Methods for a complete description of the TIGER algorithm.

To test the TIGER algorithm, we used three TF knock-out (TFKO) and two tissue- and cell-type-specific datasets (Fig. 1b). In the TFKO datasets (T1 and T2), there is a clear way to evaluate the results of the prediction algorithms: test whether the knocked-out TF has the lowest predicted activity in that sample. We also validated the structure of the inferred GRN using independent data from yeast (transcriptomic data from TF overexpression experiments) or from A375 and MCF7 cancer cell lines (ChIP-seq data on 11 of the knocked-out TFs from Cistrome DB^15^). In the third and fourth tests (T3 and T4), we evaluated TIGER’s performance in real applications, specifically focusing on normal breast tissue from the Genotype Tissue Expression (GTEx) dataset, the 10x Genomics peripheral blood mononuclear cell (PBMC) single-cell multi-omics dataset, and the paired mouse bulk multi-omics dataset from the ENCODE project. In these contexts, there is no accepted gold standard to use for evaluating the algorithm. We therefore applied TIGER to compare TFA in female and male breast tissue and examined the results manually. We then applied TIGER to a publicly available 10x Genomics single-cell multi-omics dataset consisting of 10,412 PBMCs and a mouse paired bulk multi-omics dataset from the ENCODE project. To gauge TIGER’s ability (or comparable methods) to infer TF activity from gene expression alone, we compared the predictions derived from RNA-seq data to a reference generated by multi-modal analysis of both scATAC-seq (or DNase-seq in mouse) and scRNA-seq (RNA-seq in mouse) datasets.

As comparisons for TIGER, we applied VIPER^2^, Inferelator^16^, CMF^9^, SCENIC^17^, and MLM, ULM, WSUM, GSVA, WMEAN, AUCELL from the decoupleR package^5^. These methods are appropriate comparisons for TIGER because they are commonly used to estimate TFA and they take in the same two inputs as TIGER – gene expression data and a prior regulatory network. The primary distinction between these methods lies in their ability to update the regulatory network during TF activity estimation; TIGER is the sole method that can update both the edge weight and edge signs. We applied all methods to the four test datasets and ranked the TFA estimates in each sample. To present the results in an intuitive manner, we have chosen to assign the TF with *lowest* estimated TFA in the TFKO datasets as rank 1, since our aim here is to identify the knocked-out TF. However, in the breast tissue and PBMC datasets, our goal is to identify the driver TFs so we assign the TF with *highest* activity a rank of 1.

To evaluate TIGER’s efficacy, we conducted an ablation study, systematically omitting each of its critical components: prior network structure constraint, edge sign constraint, and TFA non-negative constraint. Utilizing the yeast TFKO dataset, we assessed TIGER and its variants’ ability to identify knocked-out TFs. The results show that every component of the TIGER algorithm is essential to its performance (Supplementary Fig. 1).

### TIGER improves TF activity estimation by improving edge sign accuracy

For the yeast TFKO dataset (T1), we compared the performance of all TFA prediction methods using the same input data. The prior network was curated from yeast ChIP data^9^, and the prior edge signs were assigned by calculating whether the target gene was increased or decreased in response to overexpression of that same TF in an independent dataset^9^. All methods can utilize this sign-constrained prior network; however, TIGER incorporates the information as either hard or soft constraints. TIGER compares the edge sign from the prior network with the partial correlation between the TF and the target gene in the gene expression input data. When these two computations lead to the same sign, we assign it as a hard constraint (i.e., the distribution is half-normal). When the two computations lead to different signs, we leave it ambiguous (i.e. the prior distribution is a full normal including both positive and negative values) and allow the algorithm to learn the final edge sign. This is in contrast to CMF, Inferelator and other methods, which always enforce a hard constraint on the edge sign and do not allow the sign to be learned from the data.

**Fig. 2:**
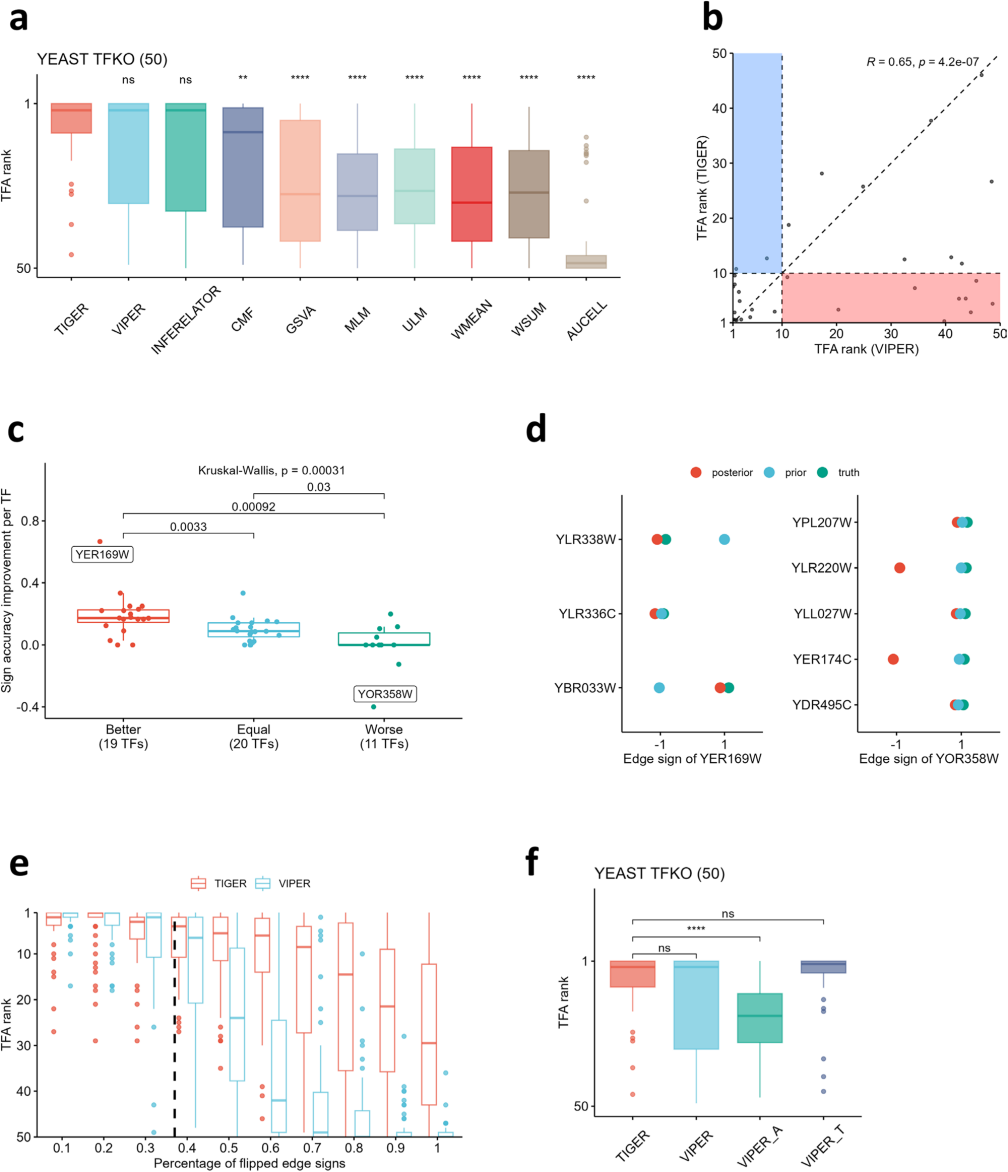
TIGER improves TF activity estimation by improving edge sign accuracy. **a** Boxplot depicting the TFA results estimated using TIGER and other methods, with pairwise comparisons to TIGER using Wilcoxon rank sum test p-values indicated above each box. Significance levels are indicated as ns (not significant), **(*p* < 0.01), and ****(*p* < 0.0001). **b** Correlation between TFA estimates from TIGER and VIPER, with a Spearman correlation coefficient of R = 0.65 (*p* < 0.05). The red rectangular region highlights the 9 TFs that TIGER successfully identifies, while VIPER fails to do so. The blue rectangular region represents 1 TF that VIPER successfully identifies, but TIGER fails to do so. (**c**) Boxplot displaying the associations between edge sign accuracy improvement (Y-axis) and TIGER’s performance (X-axis), where “Better,” “Equal,” and “Worse” correspond to TFs for which TIGER outperforms, equals, or underperforms VIPER, respectively. The Kruskal-Wallis test revealed a significance level of *p* < 0.05. The figure highlights two TFs with the maximum and minimum improvement. **d** Edge sign dot plot of YER169W (left) and YOR358W (right), with dot color indicating posterior edge sign, prior edge sign, and true edge sign. **e** Boxplot comparing TIGER and VIPER performance on the prior network with randomly flipped edge signs. The X-axis denotes the percentage of flipped edge signs ranging from 10 to 100%, with the dashed vertical line representing the actual rate (37%) of wrong edge signs in the yeast TFKO data. **f** Boxplot comparison of context-specific and generic prior networks using VIPER. VIPER_A stands for VIPER using context-specific ARACNe network. VIPER_T stands for VIPER using TIGER estimated network. TIGER is the reference and pairwise Wilcoxon rank sum test p-values are indicated as ns (not significant) or ****(*p* < 0.0001).

Considering all the TFs in the knockout dataset, we found that TIGER performs better than all other TFA estimation methods, with a slightly higher mean performance than VIPER and Inferelator, and significantly better performance than the other methods including CMF (Fig. 2a; Wilcoxon test *p* < 0.05). We further compared TIGER and VIPER in detail since they have the most similar performance. The Spearman correlation of their TFA rankings reveals a high consistency of R = 0.65 (*p* < 0.05) (Fig. 2b). However, if we define a “success” as the knocked-out TF being among the top 10 results (motivated by resource limitations to experimentally validate lower-ranked hypotheses), we observe that TIGER succeeds at identifying 40 TFs whereas VIPER succeeds at identifying 31 TFs, thus demonstrating TIGER’s improved ability to estimate TF activity.

We hypothesized that TIGER has better performance due to its ability to improve the regulatory network model. Because the prior network structure for yeast already uses high-quality ChIP data, we focused on evaluating TIGER’s ability to improve the accuracy of the edge signs. To define a “gold standard” for the edge signs, we used the change in target gene expression directly measured in the TF knockout data, rather than in the overexpression data that was used as input to the method. Comparing the total edge sign accuracy for the prior and posterior network over all TFs, we confirmed that TIGER improves this accuracy score from 0.63 to 0.73 (Supplementary Fig. 2). Then we asked whether this improvement in edge signs drives the improved performance of TIGER over VIPER. To answer this question, we sorted all TFs into three groups—“Better,” “Equal,” and “Worse”—which correspond to TIGER having a better, equivalent, or worse performance than VIPER. For each TF, we computed the edge sign accuracy improvement, defined as the posterior accuracy minus prior accuracy. We found that TIGER’s performance is highly correlated with the edge sign accuracy improvement (Fig. 2c, KW test, *p* < 0.05). We noted that the majority of TFs for which TIGER improved upon VIPER (the “Better” group) demonstrated significant improvements in edge sign accuracy, particularly TF YER169W which accurately corrected two erroneous edge signs in the prior network (Fig. 2d, left). For the small number of TFs where TIGER performed worse than VIPER (“Worse”), the edge sign accuracy showed very little or negative improvement. For instance, TF YOR358W incorrectly flipped two edge signs that were correct in the prior (Fig. 2d, right). However, these two edges have the lowest weights (Supplementary Fig. 3), showing that TIGER had low confidence in this prediction. More generally, for almost all the TFs, we observed an edge sign accuracy increase after using TIGER (Supplementary Fig. 4). Overall, these results indicate that TIGER has an advantage when the prior network is imperfect and can be improved by finding consistency with the expression data. We conducted a simulation study to quantify this phenomenon and found that TIGER starts to perform better than VIPER when more than 20% of edge signs are inaccurate in the prior (Fig. 2e). TIGER is therefore likely to provide an advantage in many contexts; for example, our yeast prior has ~37% “inaccurate” edge signs when compared with the TF knock-out data, and mammalian systems have even more limited information available on prior network signs.

Finally, we asked whether prior networks from consensus databases or computationally inferred regulons yield better results. Instead of using ChIP data to build a prior, we inferred a network directly from the yeast TFKO dataset using ARACNe and applied VIPER to estimate TFA (this workflow is labeled “VIPER_A”). We found that VIPER using the ChIP prior outperformed VIPER_A (Fig. 2f), which is in line with previous studies showing that high-confidence regulatory networks from databases lead to better performance for VIPER than context-specific inferred networks^3,4^. Moreover, we found that using the TIGER posterior network as prior information for VIPER (labeled as “VIPER_T”) exhibits better performance than VIPER using either the ChIP prior or the ARACNe prior, reinforcing the notion that combining high-confidence databases with context-specificity in a single framework improves TFA prediction.

### TIGER improves TF activity estimation by re-weighting edges

**Fig. 3:**
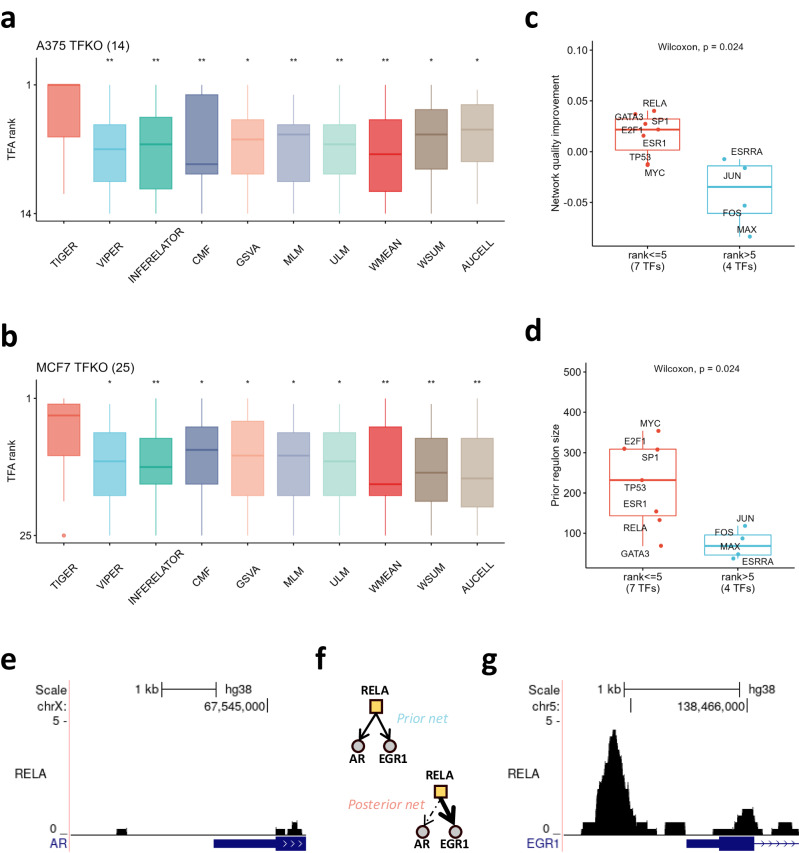
TIGER improves TF activity estimation by re-weighting edges. **a**, **b** Boxplots demonstrate the TFA results estimated from TIGER and other methods. Wilcoxon rank sum tests were used to compare all methods against TIGER, with *p* values indicated at the top of each box (ns = not significant, *p < 0.05, **p < 0.01, ***p < 0.001, ****p < 0.0001). **c** Boxplot displays the association between the regulon Quality Score (QS) improvement (Y-axis) and TIGER’s performance (X-axis), with the red box denoting the 7 TFs that TIGER successfully identifies (rank < =5) and the blue box indicating the 4 TFs that TIGER fails to identify (rank > 5). The Wilcoxon rank sum test between these two groups is labeled. **d** Boxplot illustrates the relationship between the prior regulon size (Y-axis) and TIGER’s performance (X-axis), with the red box representing the 7 TFs that TIGER successfully identifies (rank < =5) and the blue box depicting the 4 TFs that TIGER fails to identify (rank > 5). The Wilcoxon rank sum test between these two groups is labeled. (**e**) Genome browser tracks present the ChIP-seq signal of TF RELA binding to the promoter r**e**gion of gene AR in MCF7 cells. **f** Network visualization of TIGER prior and posterior edges. **g** Genome browser tracks present the ChIP-seq signal of TF RELA binding to the promoter region of gene EGR1 in MCF7 cells.

We next tested TIGER’s performance on two human cell lines. Both A375 melanoma and MCF7 breast cancer cells have TFKO transcriptomic datasets (T2), which have been used previously for TFA inference using the DoRothEA database^3^. DoRothEA is a collection of highly curated TF regulons that can serve as signed network priors for TF activity inference. Using the TFKO expression data and DoRothEA as inputs, we compared TIGER to the other methods and found that TIGER outperformed all the other methods significantly (Fig. 3a-b; Wilcoxon *p* < 0.05). We reason that TIGER shows even more improvement over VIPER in this case because cancer cell lines have more complicated regulatory mechanisms that limit the accuracy of the prior network. Additionally, the DoRothEA prior network is not specific to either of the A375 or MCF7 cell lines, meaning there could be many context-irrelevant edges.

We asked whether TIGER’s improved performance comes from its ability to re-weight edges, specifically, up-weighting true edges and down-weighting irrelevant edges. To test our hypothesis, we downloaded available ChIP-seq data for A375 and MCF7 from Cistrome DB^15^. For A375, there was ChIP-seq data available for 1 out of 14 knocked-out TFs, and for MCF7, there was data for 10 out of 25 knocked-out TFs. To assess the accuracy of the edge weights, we defined a regulon quality score (QS), representing the inner product between the model-assigned edge weights and the ChIP-seq scores (see Methods). All edges have the same weight in the prior network, meaning that the prior QS will be the arithmetic mean of all edges’ ChIP-seq scores and the posterior QS will be a weighted average of ChIP-seq scores. If TIGER correctly increases the weights of true edges (i.e., those with a higher ChIP-seq score) and decreases the weights of false edges (those with a lower ChIP-seq score), the posterior QS will be larger than the prior QS. With this definition, we found that TIGER’s success at estimating TFA was significantly correlated with regulon QS improvement (Fig. 3c; Wilcoxon test, *p* < 0.05). In particular, out of the three TFs (RELA, GATA3, and TP53) for which TIGER has better performance than VIPER, RELA and GATA3 have the largest QS improvement. However, TP53 has a decrease in the regulon quality, indicating that TIGER’s performance depends on multiple factors (Supplementary Fig. 5).

Regulon size (the number of target genes of the TF in the prior network) appears to be another factor influencing the success of both TIGER and VIPER. For example, MYC, TP53, and other TFs that were successfully identified by TIGER (“rank < =5”) have significantly larger regulon sizes than the four TFs that TIGER failed to identify (“rank > 5”) (Fig. 3d; Wilcoxon test, *p* < 0.05). VIPER and TIGER both perform better on TFs with large regulons, but TIGER appears to have more tolerance for smaller regulon size (Supplementary Fig. 6; Wilcoxon test, *p* < 0.05). For instance, the regulon size of MYC is the largest with 354 genes, whereas ESRRA has the smallest with 37 genes. TIGER and VIPER are both successful in identifying the former but not the latter. In contrast, RELA has 132 target genes, and TIGER is more successful than VIPER at estimating its activity. We also asked if performance could be related to prior regulon quality, but neither TIGER nor VIPER’s performance is correlated with the prior regulon quality (Supplementary Fig. 7).

In conclusion, we found that TIGER improves TFA estimation by integrating regulatory interactions from curated databases and then re-weighting those interactions to better fit the cell type and disease condition under study. For example, RELA is the p65 subunit of the transcription factor NFkB. According to DoRothEA, one of its high-confidence target genes (assigned confidence level “A”) is the androgen receptor gene AR. The regulation of AR by NFkB is supported by previous research confirming mutual transcriptional interference between RELA and AR^18^, and recent reports that RELA mediates IL-1 repression of AR mRNA and AR activity^19^. However, ChIP-seq data from MCF7 cells shows that RELA does not directly bind the promoter region of the AR gene in this specific cell line (Fig. 3e). Indeed, when applied to MCF7 data, TIGER successfully filtered out this interaction by decreasing the edge weight between RELA and AR so that AR ranks 128 out of the 132 target genes of RELA (Fig. 3f). In contrast, TIGER increases the edge weight between RELA and EGR1 so that EGR1 is the top-ranked target gene. Consistent with this, MCF7 cells exhibit a strong ChIP-seq signal for RELA in the promoter region of EGR1 (Fig. 3g). We further explored the binding of other TFs using MCF7 ChIP-seq data from Cistrome DB, identifying several context-specific TF bindings as depicted in Supplementary Fig. 8. For example, TIGER predicts that TP53 activates transcription of BAK1, a mitochondrial membrane protein that is known to work with TP53 to induce apoptosis, and this is reflected in the ChIP-seq peaks from MCF7 cells^20^. In this way, TIGER can learn cell-line-specific regulatory interactions from the data and use them to iteratively improve TFA estimation.

### TIGER reveals sexual dimorphism in normal breast tissue

**Fig. 4:**
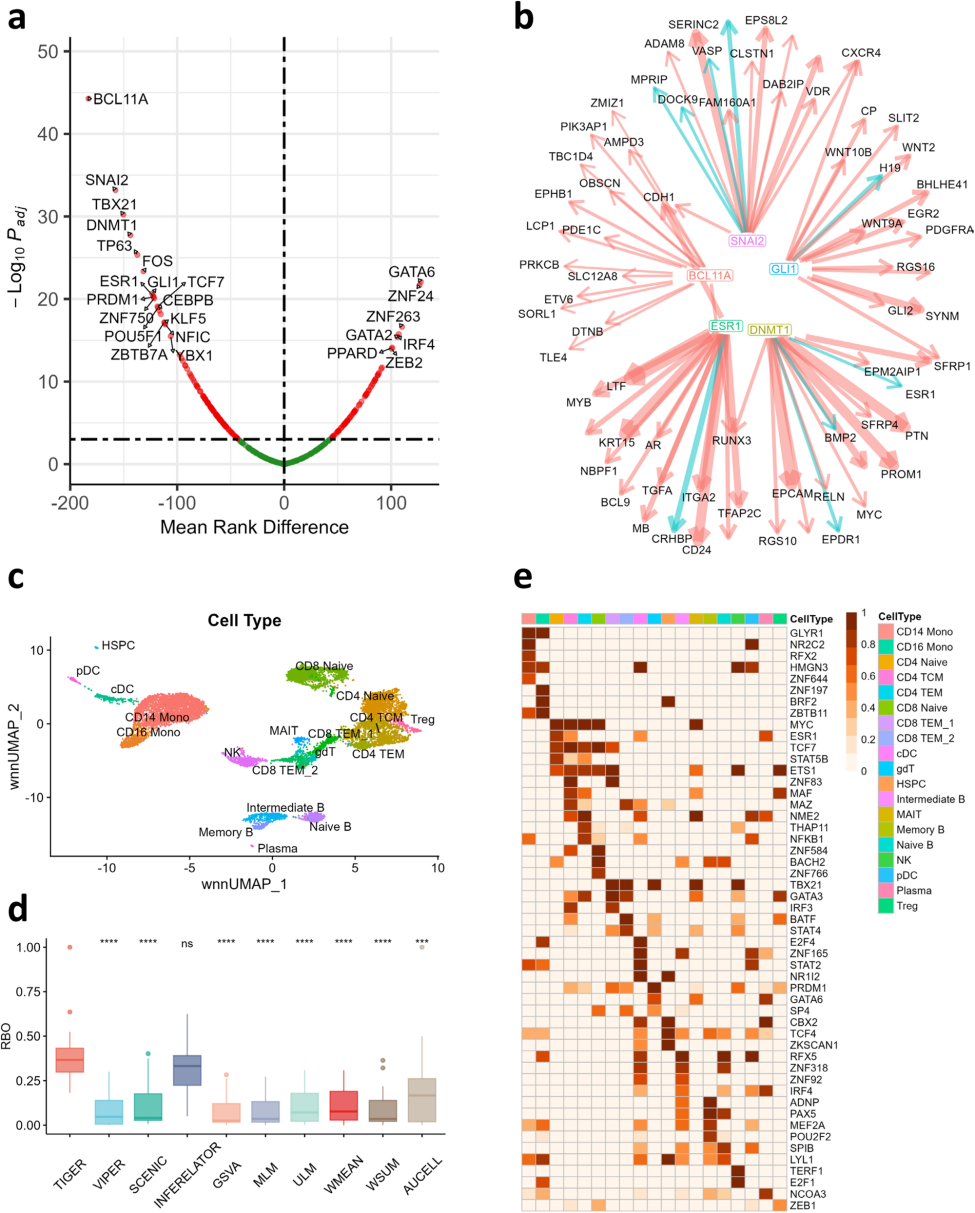
TIGER reveals tissue- and cell-type-specific regulation. **a** Volcano plot depicting differentially activated TFs in male vs. female breast tissue identified using the Wilcoxon rank sum test. Dashed lines indicate the cutoffs at adjusted *p* value = 0.001 and mean rank difference = 0. **b** TIGER network illustrating 5 active female TFs and their top 15 target genes. Activation events are denoted by red edges, while inhibition events are indicated by cyan edges. Edge width is proportional to regulation strength. **c** UMAP plot depicting 19 cell types identified by the weighted nearest neighbor algorithm. **d** Boxplots showing the RBO scores of TIGER and other methods, where a higher RBO score indicates a greater similarity to the multi-modality “gold standard.” Wilcoxon rank sum test p-values against TIGER are indicated. **e** Heatmap presenting the activity scores of the top 5 TIGER TFs in each cell type.

The GTEx consortium has conducted extensive gene expression analysis using tissues obtained from 54 different body sites, involving nearly 1000 individuals. Notably, the breast tissue exhibits the most substantial variation in autosomal gene expression between males and females^21^. We applied TIGER and other techniques to estimate sample-specific transcription factor (TF) activities in GTEx breast tissue and used a Wilcoxon rank sum test to compare TF activity between female and male individuals. TIGER identified 167 differentially activated TFs (FDR < 0.001), with the most significant ones depicted in the volcano plot (Fig. 4a). Strikingly, TIGER was the only method that placed the female hormone estrogen receptor (ESR1) among the top 10 most active TFs in females compared to males. Alongside ESR1, TIGER also identified BCL11A, SNAI2, DNMT1, and GLI1 within its top 10 TFs list for females. These TFs are known to play crucial roles in breast tissue stem cell maintenance and/or breast cancer progression^22–25^. To provide further insight, we visualized the top 15 target genes of these TFs (Fig. 4b). For instance, the primary target of ESR1 is LTF (Lactotransferrin), an estrogen-responsive gene with abundant protein production in human milk^26,27^. TIGER also reveals that DNMT1 inhibits the ESR1 gene itself, consistent with reports that DNMT1 functions as a DNA methyltransferase capable of repressing estrogen receptor expression^25^. By ranking genes based on their TIGER network in-degrees (summing all absolute weights for edges targeting a gene), we discovered that the top-ranked genes are significantly enriched in the estrogen signaling KEGG pathway (Supplementary Fig. 9; GSEA, FDR < 0.001).

### TIGER improves identification of cell type-specific TF regulators

Since TIGER is able to refine the regulatory network to reflect the cellular context, we applied TIGER and all other methods to only the scRNA-seq data from the 10x Genomics PBMC10K multiome dataset, screening out the scATAC-seq data completely, and asked which method was best able to infer TFA from the gene expression data and DoRothEA prior. Premised on the notion that different cell types can have distinct regulatory mechanisms, we applied all methods separately to each cell type curated by the 10x Genomics team. To create a “gold standard” for comparison, we used a widely accepted pipeline for inferring cell-type-specific TF activity from single-cell multiome data. To do this, we applied the Weighted Nearest Neighbor (WNN) method from “Seurat”^28^ to cluster and identify cell types using both scRNA-seq and scATAC-seq profiles (Fig. 4c). Then we used the “Signac” and “chromVAR” workflow to combine motif scores with scATAC-seq and scRNA-seq data in each cell type to estimate the most active TFs, i.e. the TFs that are actively binding to accessible promoters of expressed target genes in that cell type^29^. We then used this gold standard to benchmark the performance of TIGER and other methods. To compare performance, we used the rank-biased-overlap (RBO) metric to quantify the similarity between multiple rankings of TFA. The RBO metric correlates two ranked vectors with more weight at the top of the ranked list where driver TFs should be found (see Methods for more details).

Overall, we found that TIGER’s TF rankings had higher consistency with the multi-modality “gold standard” than most other methods (Fig. 4d; Wilcoxon Test, *p* < 0.05). The Inferelator exhibited comparable performance with TIGER. CMF was not included in this comparison because it is not optimized for large-scale datasets and resulted in an out-of-memory issue even with 500GB RAM. Instead, we employed SCENIC, a widely recognized single-cell TFA estimation method, for comparative analysis. The lower RBO for SCENIC may be due to the fact that SCENIC was designed to be applied to the full PBMC dataset, whereas TIGER infers separate networks for each cell cluster. We visualized the top 5 TFs predicted by TIGER in each cell type using a heatmap (Fig. 4e) and identified several TFs with evidence in the literature for their relevance to that cell type (Supplementary Table 1). For example, IRF4 is known to control differentiation in various immune cell types, but is not as critical for memory B cells^30–32^. TIGER uniquely identifies its high activity in intermediate/naïve B cells and dendritic cells but not in memory B cells (Supplementary Fig. 10). Similar patterns are observed for other TFs like PAX5 in memory B cells^32^ (Supplementary Fig. 11), TBX21 in NK cells^33^ (Supplementary Fig. 12), and TCF4 in DC and monocytes^34,35^ (Supplementary Fig. 13). In general, we observed that, although the naive mRNA expression of the TF, the multi-modality “gold standard” TF activity, and the TIGER predicted TF activity are often correlated with each other, TIGER can sometimes uncover TF activity that cannot be observed using other methods. To assess the cell type-specific networks generated by TIGER, we ranked genes by their in-degree, selecting the top 50 for over-representation analysis in the KEGG database. This revealed enrichment in many cell type-specific pathways, such as PI3K-Akt signaling and Th17 cell differentiation in Treg cells (Supplementary Fig. 14), B cell receptor signaling in both Naïve and Memory B cells (Supplementary Figs. 15-16), and T cell receptor signaling in Gamma delta T cells (Supplementary Fig. 17).

Lastly, we extended our evaluation of TIGER to paired bulk RNA-seq and DNase-seq data from mouse cell lines in the ENCODE project. Employing a similar approach to that used with the PBMC single-cell multi-omics dataset, we identified TFs enriched in RNA-seq measurements and with increased motif accessibility in DNase-seq measurements, designating these as our ground-truth TFs. For further details, refer to our Methods section. TIGER performed better than most alternative methods, with AUCELL being the only method that showed comparable performance (Supplementary Fig. 18).

## Discussion

We present TIGER, a Bayesian matrix factorization method to better estimate regulatory networks and TF activity (TFA) by incorporating prior knowledge and gene expression data. Compared to other methods, TIGER has three main advantages. First, it distinguishes activation and inhibition events by flexibly incorporating prior edge signs and updating them from the expression data. Second, it up-weights essential edges and shrinks irrelevant edges towards zero through a sparse Bayesian prior. Third, it simultaneously estimates TFA and the regulatory network, thus obviating the difficult task of selecting the optimal combination of network inference methods and TFA estimation methods.

Our study revealed that TIGER surpasses multiple TFA estimation methods across three validation datasets. VIPER is efficient for estimating TF activity from small numbers of samples. TIGER, on the other hand, leverages a larger dataset with a common underlying regulatory model (e.g. a cohort of patients with similar disease phenotypes or a cluster of single cells annotated to the same cell type) and is able to improve TFA estimation. Consistent with previous findings, we observed that VIPER has better performance when using a set of high-confidence regulatory interactions like DoRothEA. We then demonstrated that TIGER outperforms VIPER on both yeast and mammalian datasets, regardless of whether VIPER is paired with static databases like DoRothEA or context-specific regulomes inferred using ARACNe. Our results indicate that context specificity is indeed advantageous when estimating TF activity, but only if it is incorporated into a framework that can manage the noise inherent in computationally inferred interactions.

To further explore what drives the improved performance of TIGER, we performed two types of analyses. In the yeast RNA-seq data, we assessed TIGER’s ability to infer correct edge signs. In the cancer cell line data, we used cell-line-specific ChIP-seq data and assessed TIGER’s ability to refine the edge weights. Our results indicate that both edge sign and edge weight are important for correct TFA estimation, and that edge sign has a high impact – despite the fact that it is sometimes ignored by network inference algorithms. TIGER is especially valuable when the accuracy of prior edge signs falls below 80% (as is often the case in real data) because it can then update and improve the signs accordingly. Our applications of TIGER to GTEx and 10x Genomics datasets suggest that TIGER can also uncover biological mechanisms in tissue-specific and cell-type-specific data.

The performance of TIGER is limited by certain features of the algorithm. TIGER takes as input a prior TF-gene network with associated edge signs; however, information on edge signs can be limited depending on the organism and system under study. For human datasets, the DoRothEA database includes curated edge sign information. For users interested in using their own prior network but lacking edge sign data, the correlation or partial correlation between TF and target gene expression could be used along with an appropriate threshold to populate hard (half-normal distribution) and soft (full normal distribution) constraints.

In the yeast TF knock-out dataset, we noticed that TIGER’s Bayesian framework attempts to update the network even if the prior network sign is correct, which can add noise to the network and decrease TIGER’s performance. One could fully constrain all edge signs to be the same as the signs in the prior network, but this would damage performance when the prior edge signs are inaccurate. An alternative would be to construct a Bayesian credible interval for every edge. If the credible interval excludes zero, we accept the new edge sign; otherwise, we keep the prior edge sign or perhaps shrink the edge weight to zero. An appropriate cutoff for the credible interval (e.g., 90% CI) could be determined by constraining the density of the regulon and the overall graph.

The number of target genes of a TF in the prior network – or regulon size – can also affect the performance of TIGER. In the cancer cell line data, we observed that TFA estimation by TIGER and VIPER relies on having a reasonably large and accurate set of targets for each TF. Although TIGER can refine edge weights, it is inherently a Bayesian filtering framework. It is limited to using the interactions provided by the prior network and is unable to learn new regulatory interactions. This can be a significant limitation, especially for TFs that are not well-studied in the literature. One possible solution is to add more computationally predicted edges into the prior network, and allow TIGER to choose the interactions that best support the expression data. To do this efficiently, the TIGER algorithm will need to be optimized to keep computational time low while avoiding overfitting. Other possibilities are to use a different prior distribution or tune hyperparameters to accommodate smaller regulons.

The model framework underlying TIGER has several limitations as well. First, it does not account for non-linear TF-TF interactions arising from competitive or cooperative binding. Second, epigenetic effects on the susceptibility of each gene to regulation, for example, by making the promoter more or less accessible to TFs, are not considered. Third, the linear model for TFA underlying TIGER cannot model saturation effects – i.e., a situation where TFA increases past a certain threshold and the expression of the target gene levels off. Fourth, TIGER is not specifically tuned for use with scRNA-seq data, and in particular is not optimized for use with sparse expression matrices and potential dropout events. To extend TIGER’s modeling capabilities, TF-TF interactions could be incorporated by integrating protein-protein interaction (PPI) information into the covariance of the TFA matrix $Z$. To address the second problem, recent work^16^ suggests that one could refine the prior networks by incorporating ATAC-seq data. For the third issue, we could use a logistic function to transform the linear combinations of TFA. The saturation of gene expression would then be reflected by the upper asymptotes. To address the dropout issue prevalent in scRNA-seq data, numerous statistical methods have emerged in recent years. Two notable approaches include the utilization of a hurdle model^36^ and the application of a left-truncated Gaussian mixture model^37^, both of which offer effective solutions to mitigate this challenge. We leave these questions for future study.

The chosen evaluation metric, e.g. the rank of the knocked-out TF, has its limitations, primarily because it focuses on a single TF and overlooks the performance across all other TFs. An alternative metric is the Spearman correlation between inferred TFA and the gene expression of the corresponding TFs (Supplementary Fig. 19). These correlations are small but positive, and there is no significant difference in correlation between TIGER and alternative methods. This suggests that TIGER’s improved performance is due to its ability to infer protein-level activities of transcription factors.

In summary, we have developed a Bayesian matrix factorization method that takes as input multiple expression profiles and jointly infers context-specific regulatory networks and TF activity levels. When applied to yeast and human RNA-seq data, TIGER outperforms other methods that are less flexible when it comes to incorporating prior knowledge on sign of regulation or cell-type specificity. Future work will continue to improve this method by integrating other biologically realistic features of transcriptional regulation and complementary data types.

## Methods

### TIGER algorithm

TIGER is based on Bayesian matrix factorization. The underlying assumption is that each gene expression profile ${X}_{n}$ for sample *n* can be modeled as ${X}_{n}={W}{Z}_{n}+\epsilon$, where $W$ is a *g* x *h* matrix of *g* genes and *h* transcription factors that represents the adjacency matrix of a weighted regulatory network and ${Z}_{n}$ is the vector of TF activities (TFA) in that sample. We use a Gaussian inverse gamma prior to sparsely encode the edge weights (the entries of $W$) if prior knowledge (${W}^{0}$) confirms that some TF $i$ is regulator of gene $j$, and a multivariate half-normal distribution for the non-negative TFA sample ${Z}_{n}$.

1 $$\left\{\begin{array}{c}{W}_{{ij}} \sim N(0,\,{\alpha }_{{ij}}\,),\,{\alpha }_{{ij}} \sim {IG}\left({a}_{\alpha },\,{b}_{\alpha }\,\right),{ if\ }{W}_{{ij}}^{0}\,\ne\, 0\\ {W}_{{ij}}=0,{ if\ }{W}_{{ij}}^{0}=0\end{array}\right.$$

2 $${{\rm{Z}}}_{{\rm{n}}} \sim {{\rm{MVN}}}^{+}\left(0,{\Sigma }_{{\rm{Z}}}\right)$$

When prior information on the edge signs is available, we apply sign constraints to the distribution of well-supported edges. Well-supported edges are identified by comparing the prior edge sign with the partial correlation between the expression of the gene encoding the transcription factor (TF) and the expression of the target gene, using the GeneNet R package. If the two signs are consistent, we add a sign constraint to the edge weight distribution by making it half-normal. However, if the two edge signs are inconsistent, the edge sign remains unconstrained, and the prior distribution is a full normal that includes both positive and negative values, which allows TIGER to automatically learn a sign based on what best fits the data. Prior to analysis, the expression matrix $X$ must be normalized and log-transformed. Thus, the error term is assumed to follow a normal distribution with a variance ${\sigma }_{\epsilon }^{2}$ following an inverse gamma distribution,

3 $${\sigma }_{\epsilon }^{2}\, \sim {IG}({a}_{\sigma },\,{b}_{\sigma })$$

By default, hyperparameters are set as ${a}_{\alpha }={b}_{\alpha }=1,\,{a}_{\sigma }={b}_{\sigma }=1,{and}\,{\sigma }_{Z}^{2}=100$. Parameter estimation is performed by either MCMC or Variational Bayes methods with a mean-field Gaussian distribution family, as implemented in the Automatic Differentiation Variational Inference (ADVI) algorithm in the probabilistic programming platform STAN^38^. The posterior mean is used to summarize $W$ and $Z$. Model checking is performed using cross-validation to estimate out-of-sample predictive accuracy. For a complete description of the TIGER model, including issues of identifiability, model constraints, and comparisons of simulation results between MCMC and Variational methods, please refer to the Supplementary Methods.

### Testing Datasets

Yeast datasets were downloaded from^9^, including TF knock-out, TF over-expression, and ChIP data. Expression data were already log-transformed and normalized, and we computed gene-wise Z-scores for input into TIGER and other methods. High-quality ChIP data were preselected based on the criterion in^9^, yielding a network of 1104 edges between 50 TFs and 778 genes. Edge signs were inferred by comparing knock-out samples with wild-type samples.

A375 and MCF7 TFKO cell line data were downloaded from^3^. Data were already log-transformed and normalized, and we computed gene-wise Z-scores for input into TIGER and other methods. Low-quality TFKO samples were excluded from the analysis based on the criterion in^3^, yielding 14 TFKO samples in A375 and 25 TFKO samples in MCF7. The DoRothEA cancer prior network “dorothea_hs_pancancer” from “dorothea” R package 1.6.0 was used. We included all A-E level DoRothEA edges. These edges are curated from many resources, such as literature databases, ChIP-seq data, TF binding sites, and ARACNe networks inferred from The Cancer Genome Atlas (TCGA)^3^. ChIP-seq validation datasets for the 11 TFs were downloaded from Cistrome DB^15^.

We obtained GTEx breast tissue data from the Recount3 database^39^ through the R package “recount3” version 1.10.2. Subsequently, we performed normalization on the gene expression data using the “getTPM” function from the R package “recount” version 1.26.0. We applied the “filterByExpr” function from the R package “edgeR” version 3.42.2 with default parameters to exclude low-expression genes. Additionally, we removed duplicate samples and scrutinized for potential batch effects, but our examination of the PCA plot did not reveal any discernible batch effects.

PBMC scRNA-seq and scATAC-seq data were downloaded from the 10X Genomics website (PBMCs from C57BL/6 mice (v1, 150 × 150), Single Cell Immune Profiling Dataset by Cell Ranger 3.1.0, 10x Genomics, 2019, July 24). Data preprocessing, weighted nearest neighbor integration, clustering, annotation, motif analysis, and driver TF identification was performed as described in the “Weighted Nearest Neighbor Analysis” vignette, updated on August 30, 2021. Specifically, R packages “Seurat” version 4.1.1, “Signac” version 1.7.0, “EnsDB.hsapiens.v86” version 2.99.0, “chromVAR” version 1.16.0, “JASPAR2020” version 0.99.10, “TFBSTools” version 1.32.0, “motifmatchr” version 1.16.0, “BSgenome.Hsapiens.UCSC.hg38” version 1.4.4, “presto” version 1.0.0 were used. TIGER and VIPER were applied on each cell type using the “dorothea_hs” prior network from “dorothea” R package 1.6.0. Because scRNA-seq data is very sparse, in each cell type, we only considered the marker genes identified using the “presto” R package with a p-value cutoff of 0.01 and log-fold-change cutoff of 0, the same as in the vignette.

Mouse cell line paired RNA-seq and DNase-seq data were sourced from the ENCODE project, focusing on 11 samples from mouse embryonic stem cells – G1E and G1E-ER4. The DNase-seq data processing utilized the “rtracklayer” 1.60.1 and “BSgenome.Mmusculus.UCSC.mm10” 1.4.3 R packages for sequence peak extraction. We used “TFBSTools” 1.38.0 and “JASPAR2020” 0.99.10 R packages to assemble mouse TF motif matrices. Through iteration over motifs and peak sequences, the “matchPWM” function from the “Biostrings” R package 2.68.1 identified TFs binding to open chromatin regions, with TFs scored by their binding frequency to these regions. Ground-truth TFs were determined based on high gene expression levels and extensive open chromatin region binding. The ground-truth TF ranking was computed by taking the average of ranked gene expression levels and open chromatin binding scores. This was compared with TFA estimate ranks using the rank-based overlap. TFA estimation was carried out using log2(TPM + 1) values from the RNA-seq data.

### VIPER

VIPER is a statistical test for the enrichment of each regulon based on the average ranks of the target genes^2^. It takes into account the sign of each TF-target interaction. We used the R package “VIPER” version 1.28.0. We used VIPER with default parameters, except the minimal regulon size filter was set to 0 to ensure no TFs will be removed in VIPER computation.

### Inferelator

Inferelator is a method for gene regulatory network inference that is based on regularized regression. For our bulk and single-cell experiments, we utilized Inferelator 3.0^16^. We employed the “BBSR” (Bayesian Best Subset Regression) and “TFA” workflow as we were interested in estimating TF activities.

### CMF

Constrained Matrix Factorization is a new variant of Network Component Analysis (NCA) that constrains the edge sign. We used the “TFAinference.py” python code v2.0.1 downloaded from^9^. Within 20 steps of iterations, the bilinear optimization algorithm converged in the three TFKO datasets we tested. However, in the normal tissue and PBMC10K dataset, the algorithm exhibits a high memory usage, resulting in out-of-memory errors even with a RAM capacity of 500GB.

### SCENIC

SCENIC, a method for inferring gene regulatory networks at the single-cell level, was utilized through the pySCENIC version 0.12.1 docker image. This approach employs GRNBoost2 for the initial estimation of gene regulatory networks, with further refinement and filtering provided by the cistarget database. Subsequently, TF activities were estimated using AUCELL, a component of the SCENIC suite designed to assess the activity of transcription factors within individual cells based on the regulatory network inferred.

### decoupleR

The decoupleR package v2.0.1 offers a range of functions, including ULM (Univariate Linear Model), MLM (Multivariate Linear Model), WSUM (Weighted Sum), GSVA (Gene Set Variation Analysis), WMEAN (Weighted Mean), and AUCELL wrapper utilized. We ran all the methods using default parameters as recommended by the authors of decoupleR.

### Regulon quality score

We defined a regulon Quality Score (QS) for each TF $i$ as an inner product between the edge weights ${W}_{{ij}}$ and the ChIP-seq scores ${C}_{{ij}}$, where $j$ indexes the target genes in the regulon of TF $i$.

4 $${{\rm{QS}}}_{{\rm{i}}}=\mathop{\sum }\limits_{j}\left({{\rm{|}}W}_{{ij}}{\rm{|}}* {C}_{{ij}}\right),{where}\mathop{\sum }\limits_{j}{{\rm{|}}W}_{{ij}}{\rm{|}}=1$$

The ChIP-seq scores ${C}_{{ij}}$ are normalized to rank percentile. For the prior network, ${W}_{{ij}}=1/n$. The Quality Score for the prior network is therefore the average ChIP-seq score of all targets. If TIGER up-weights the true edges and down-weights the irrelevant edges, the posterior QS will increase. Thus, we defined the quality improvement as (Posterior QS – Prior QS)/Prior QS.

### RBO similarity

Rank-biased-overlap is used to evaluate two ranked lists based on the formula from “A Similarity Measure for Indefinite Rankings”^40^. Two ranking vectors with high RBO scores are similar especially at the top-ranked portions of the vectors. The RBO value can range between 0 and 1. We used the R package “gespeR” version 1.26.0 to compute RBO similarity.

### ARACNe

ARACNe networks were constructed using the ARACNe-AP algorithm^41^. One hundred steps of bootstrap were used with *p*-value < 1E-8 as the cutoff.

### Pathway enrichment analysis

For the pathway enrichment analysis, all target genes in the network were ranked according to their in-degree (i.e., the sum of the absolute value of all edges targeting that gene), and the clusterProfiler R package 4.8.3 and the org.Hs.eg.db R package 3.17.0 were employed to conduct gene set enrichment analysis (GSEA) and over-representation analysis (ORA).

### Reporting summary

Further information on research design is available in the Nature Research Reporting Summary linked to this article.

### Supplementary information

Supplementary Information

Reporting summary

## Data Availability

All the testing datasets are available on Zenodo (10.5281/zenodo.7425777).

## Code availability

The TIGER package is available on GitHub (https://github.com/cchen22/TIGER) and now a part of netZooR v1.4 package (https://github.com/netZoo/netZooR)^42^. The analysis codes to reproduce the results and figures are available on Zenodo (10.5281/zenodo.7425777).
