# Supplementary material for: Flexible modeling of regulatory networks improves transcription factor activity estimation
Source: NPJ Syst Biol Appl. 2024 May 28;10:58. doi: 10.1038/s41540-024-00386-w (PMC11133322; doi:10.1038/s41540-024-00386-w)
Supplement: Supplementary file 1 — Supplementary Information [file 41540_2024_386_MOESM1_ESM.pdf]

SUPPLEMENTARY INFORMATION

Chen Chen
Department of Epidemiology and Biostatistics
University of Arizona
Tucson, AZ 85719
cchen22@arizona.edu

Megha Padi
Department of Molecular and Cellular Biology
University of Arizona
Tucson, AZ 85719
mpadi@arizona.edu

Contents

1 Supplementary Figures 1-19 **2**

2 Supplementary Table 1 **22**

3 Supplementary Methods **24**

3.1 The TIGER Model 24
3.2 Identifiability Issue 24
3.3 Model Constraints 24
3.4 Bayesian Framework 24
3.4.1 Prior distributions 25
3.4.2 Likelihood 25
3.4.3 Parameter estimation 25
3.4.4 Model checking 26
3.4.5 Software 26
3.5 Sign constraints revisit 26

1 Supplementary Figures 1-19

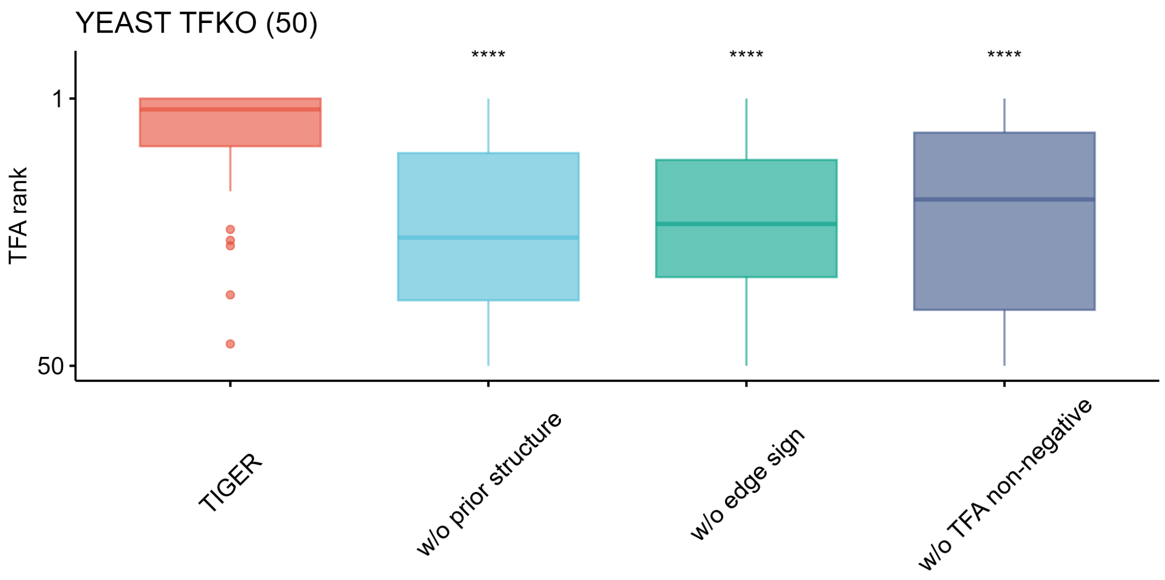

Supplementary Figure 1. Evaluation of TIGER through an ablation study. The study systematically removes key components: the prior network structure, the prior edge sign, and the non-negative constraint applied to the transcription factor activity (TFA) matrix. In each case, TIGER demonstrates superior performance compared to the versions lacking these individual elements. Related to Figure 1.

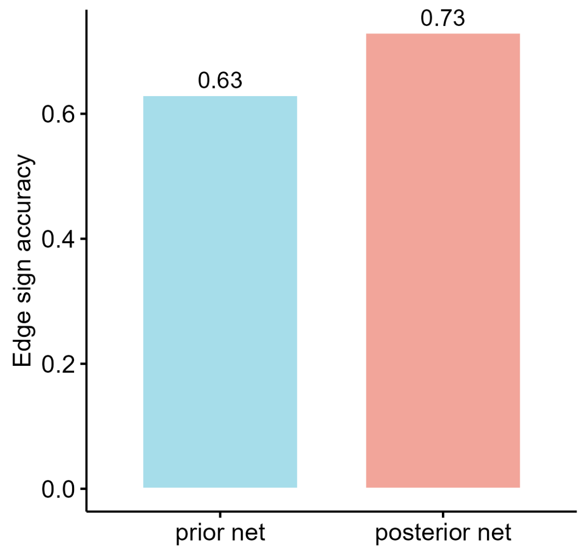

Supplementary Figure 2. Sign accuracy improves from 0.63 to 0.73 using TIGER on the Yeast dataset. Related to Figure 2.

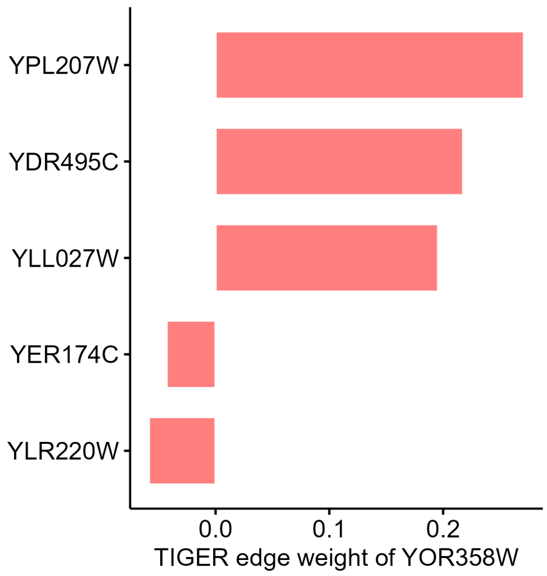

Supplementary Figure 3. Five weighted targets of YOR358W. Related to Figure 2.

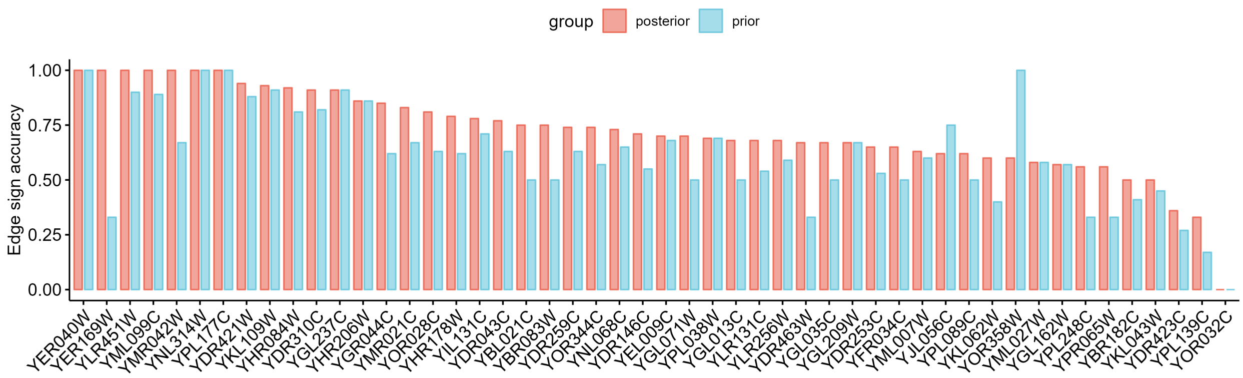

Supplementary Figure 4. Prior and posterior edge sign accuracy for each TF. TIGER correctly flips most of the edge signs except YJL056C and YOR358W. Related to Figure 2.

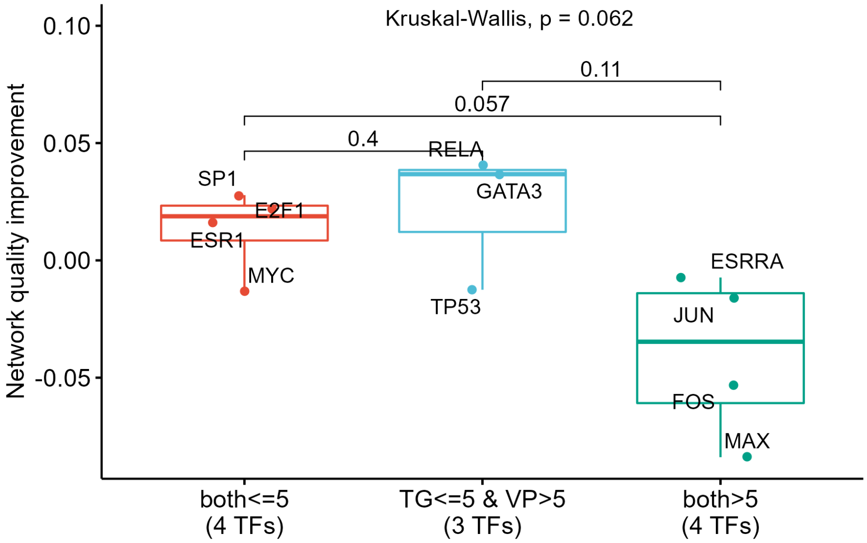

Supplementary Figure 5. Association between network quality improvement (y-axis) and the performance of TIGER (VIPER) (x-axis). Related to Figure 3. TFs are divided into three groups: "both<=5" represents TFs that are well-ranked by both TIGER and VIPER. "TG<=5 & VP>5" represents TFs that are successfully identified by TIGER but not by VIPER. "both>5" represents TFs that are not effectively identified by either TIGER or VIPER.

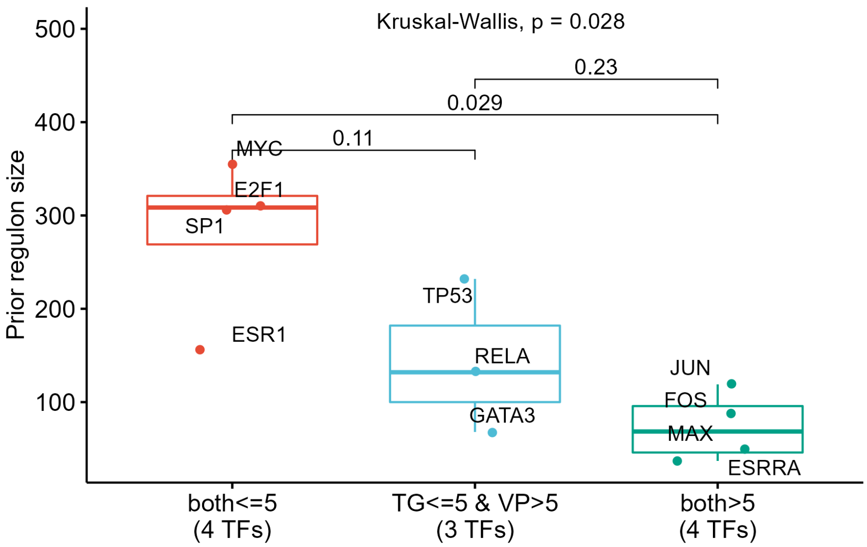

Supplementary Figure 6. Association between prior regulon size (y-axis) and the performance of TIGER (VIPER) (x-axis). Related to Figure 3. TFs are divided into three groups: "both<=5" represents TFs that are well-ranked by both TIGER and VIPER. "TG<=5 & VP>5" represents TFs that are successfully identified by TIGER but not by VIPER. "both>5" represents TFs that are not effectively identified by either TIGER or VIPER.

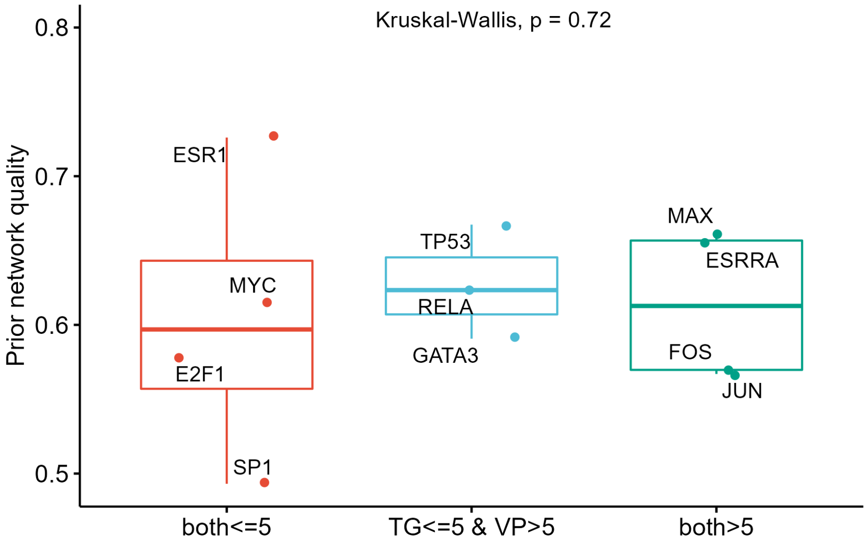

Supplementary Figure 7. Association between prior regulon quality (y-axis) and the performance of TIGER (VIPER) (x-axis). Related to Figure 3. TFs are divided into three groups: "both<=5" represents TFs that are well-ranked by both TIGER and VIPER. "TG<=5 & VP>5" represents TFs that are successfully identified by TIGER but not by VIPER. "both>5" represents TFs that are not effectively identified by either TIGER or VIPER.

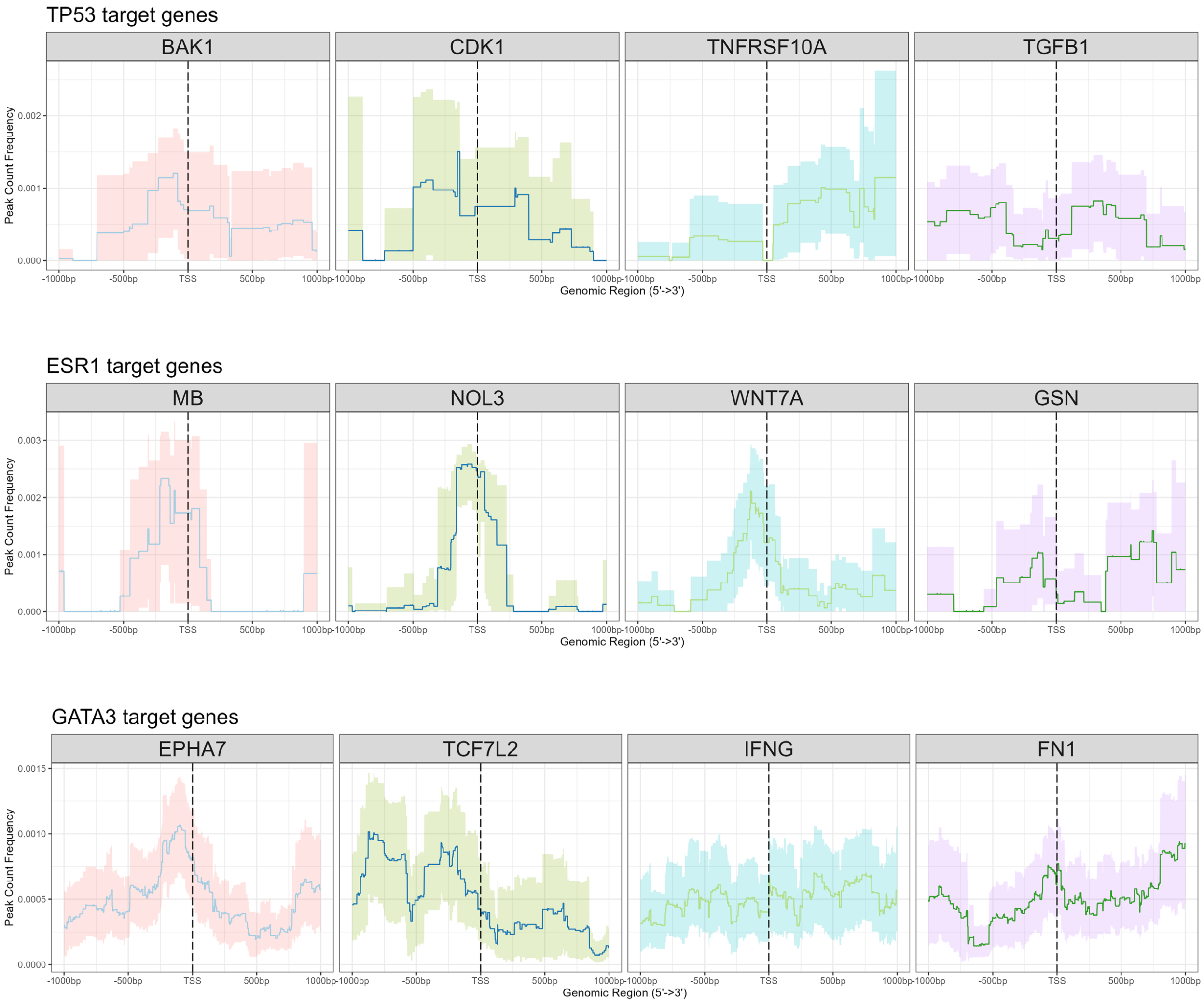

Supplementary Figure 8. ChIP-seq peak profiles of TP53, ESR1, and GATA3 in gene promoters in MCF7 cells. Each row represents a distinct TF. The two columns on the left display target genes with high edge weights as predicted by TIGER, aligning with strong ChIP-seq peak signals. Conversely, the two columns on the right show target genes with low edge weights as predicted by TIGER, which reflects the weaker ChIP-seq signals. Related to Figure 3.

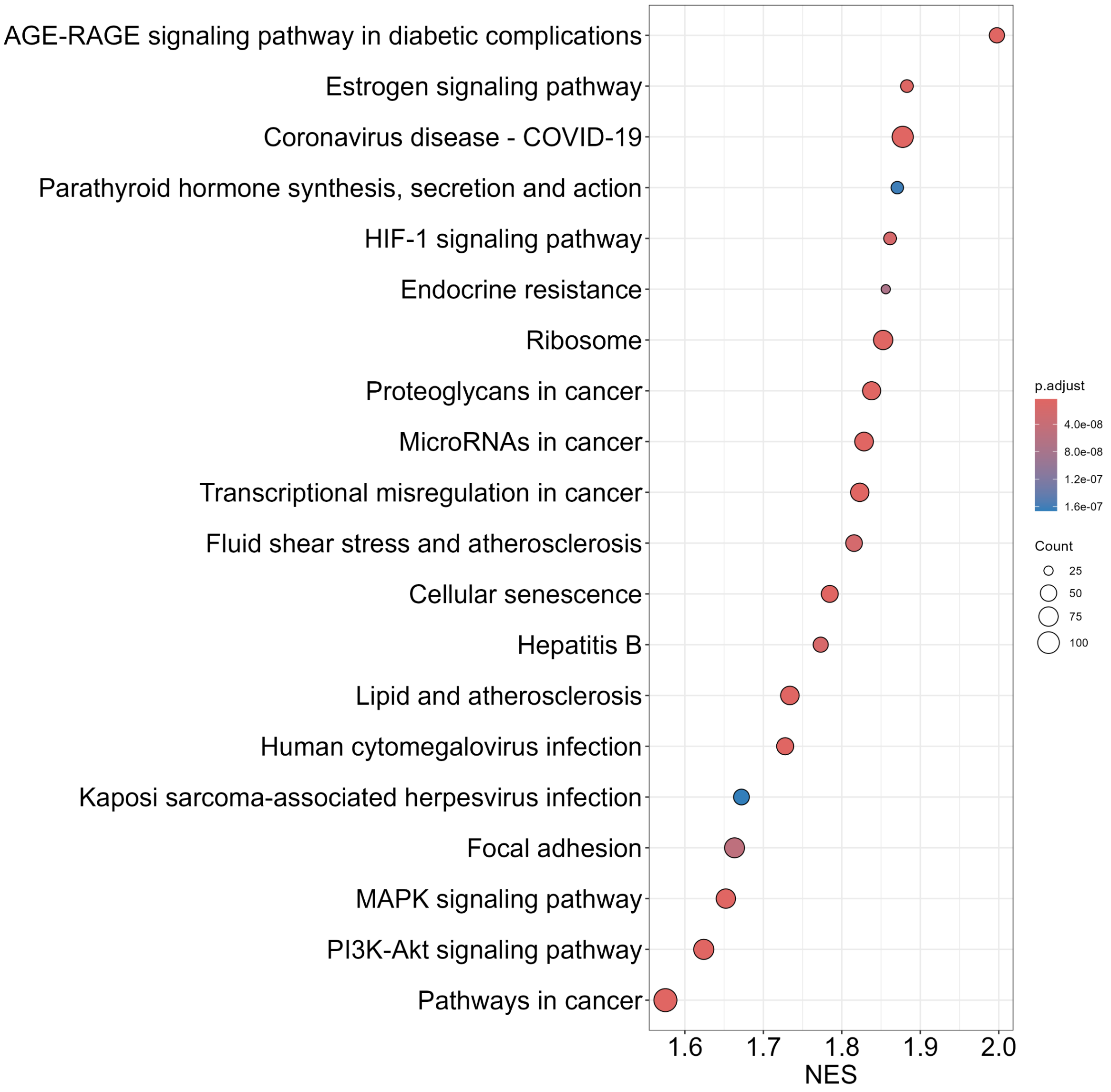

Supplementary Figure 9. Enriched KEGG Pathways in Breast Tissue. Genes were ranked using node degrees from a TIGER-estimated Gene Regulatory Network (GRN). The Gene Set Enrichment Analysis (GSEA) algorithm, in conjunction with the KEGG pathway database, was utilized to identify pathways with significant enrichment. The diagram illustrates the top 20 pathways that exhibit the highest degree of enrichment. Related to Figure 4.

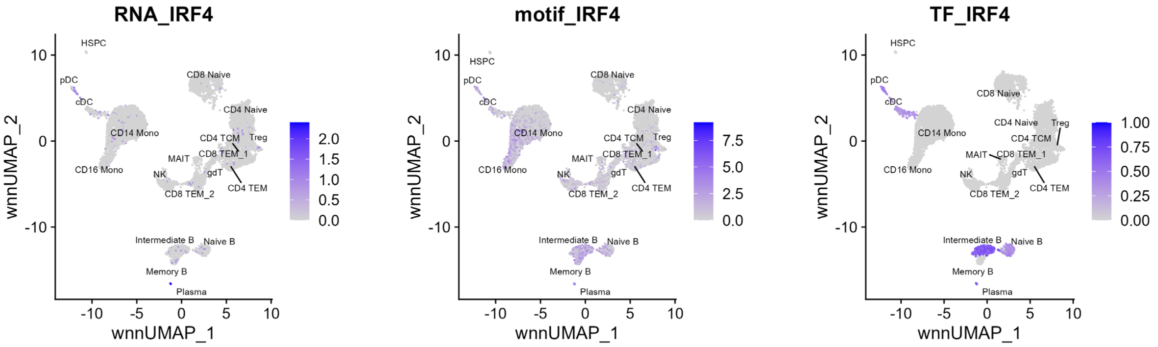

Supplementary Figure 10. IRF4 mRNA expression, motif score, and TFA level in different cell types. Related to Figure 4.

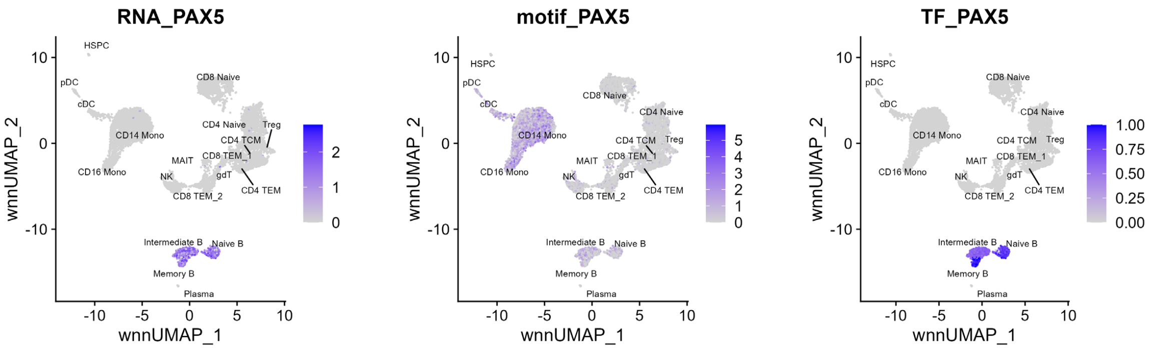

Supplementary Figure 11. PAX5 mRNA expression, motif score, and TFA level in different cell types. Related to Figure 4.

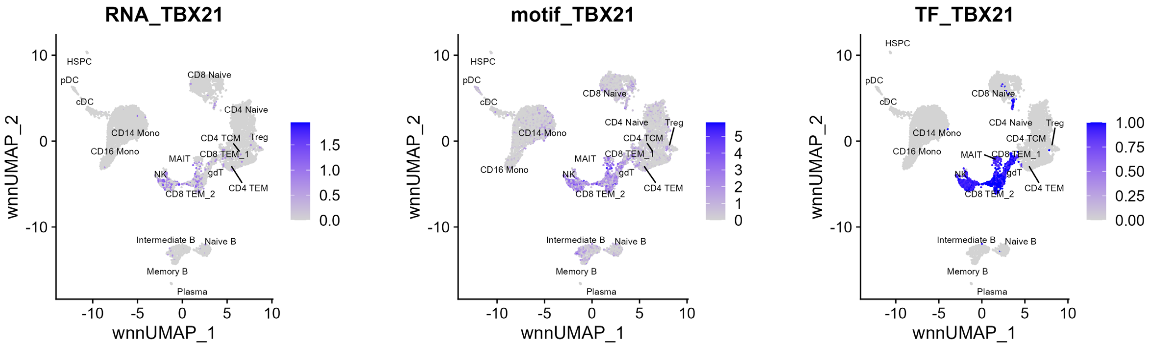

Supplementary Figure 12. TBX21 mRNA expression, motif score, and TFA level in different cell types. Related to Figure 4.

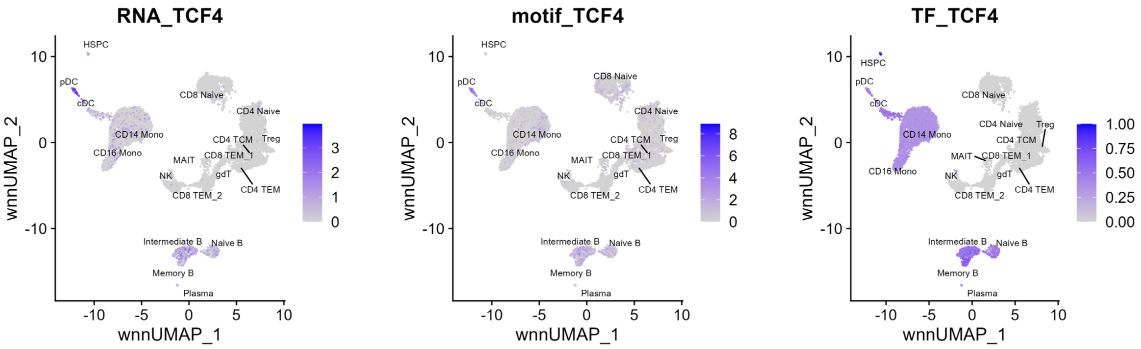

Supplementary Figure 13. TCF4 mRNA expression, motif score, and TFA level in different cell types. Related to Figure 4.

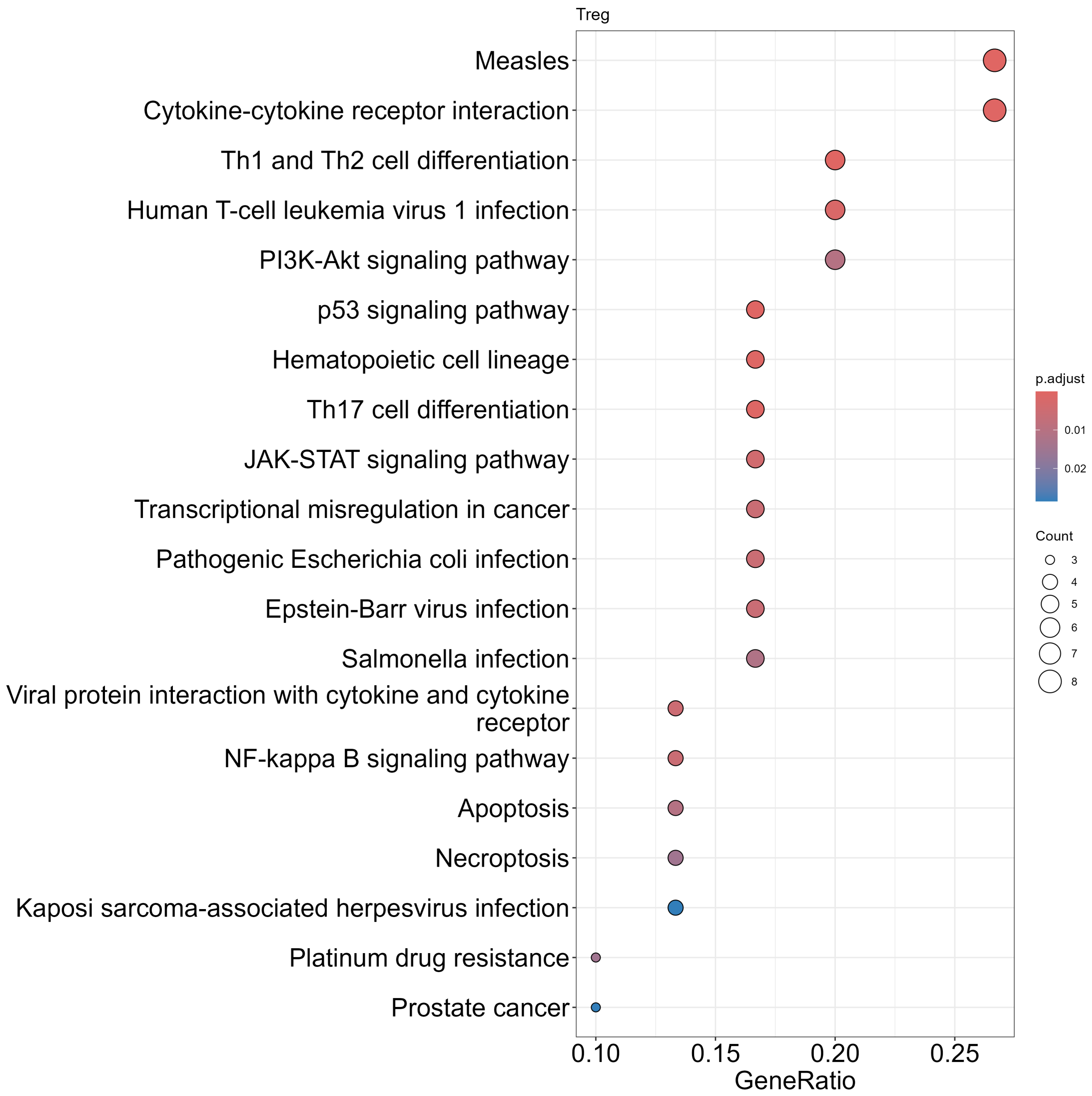

Supplementary Figure 14. Enriched KEGG Pathways in Treg cells. Genes were ranked using node degrees from a TIGER-estimated Gene Regulatory Network (GRN). Over representation analysis on top 50 genes in conjunction with the KEGG pathway database, was utilized to identify pathways with significant enrichment. Related to Figure 4.

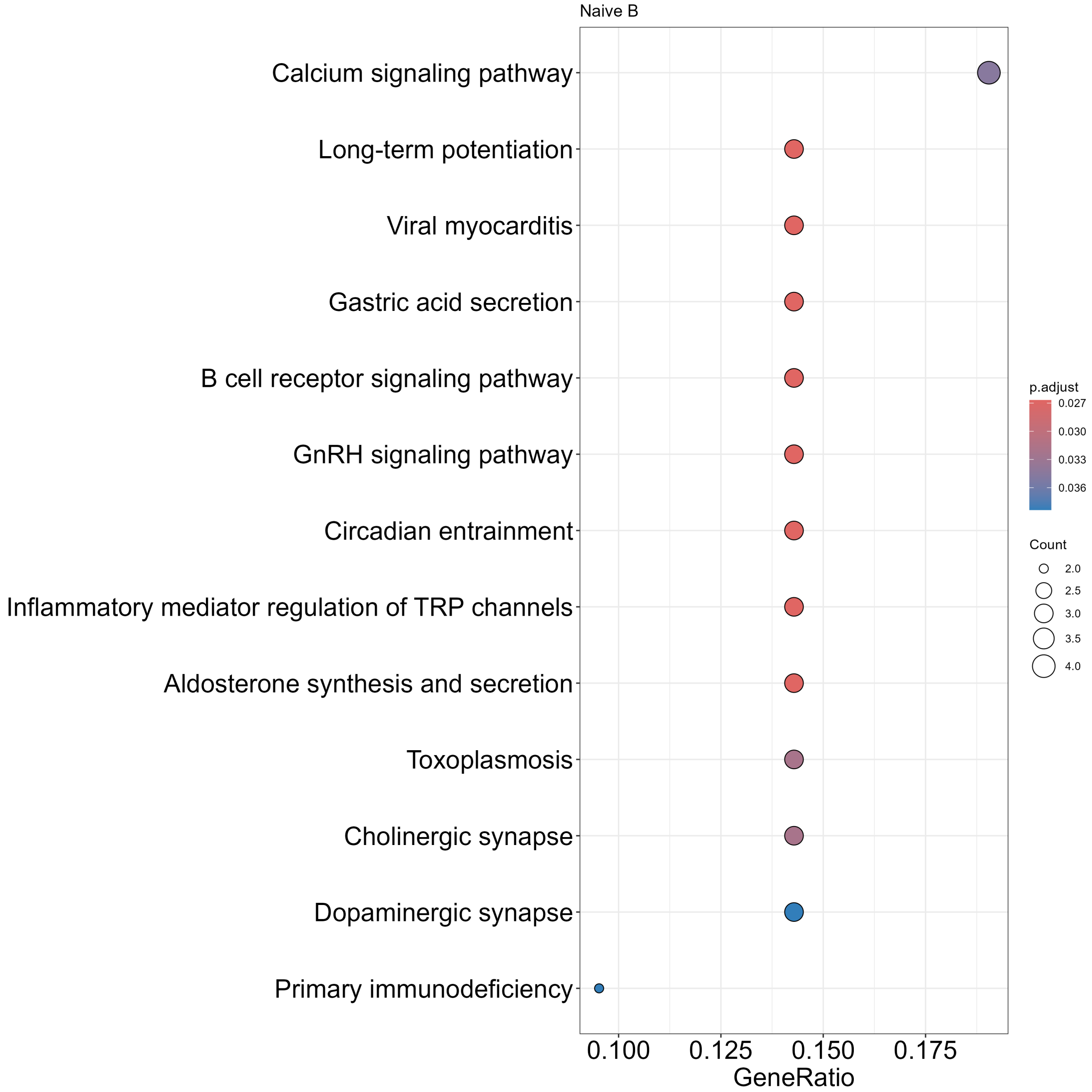

Supplementary Figure 15. Enriched KEGG Pathways in Naïve B cells. Genes were ranked using node degrees from a TIGER-estimated Gene Regulatory Network (GRN). Over representation analysis on top 50 genes in conjunction with the KEGG pathway database, was utilized to identify pathways with significant enrichment. Related to Figure 4.

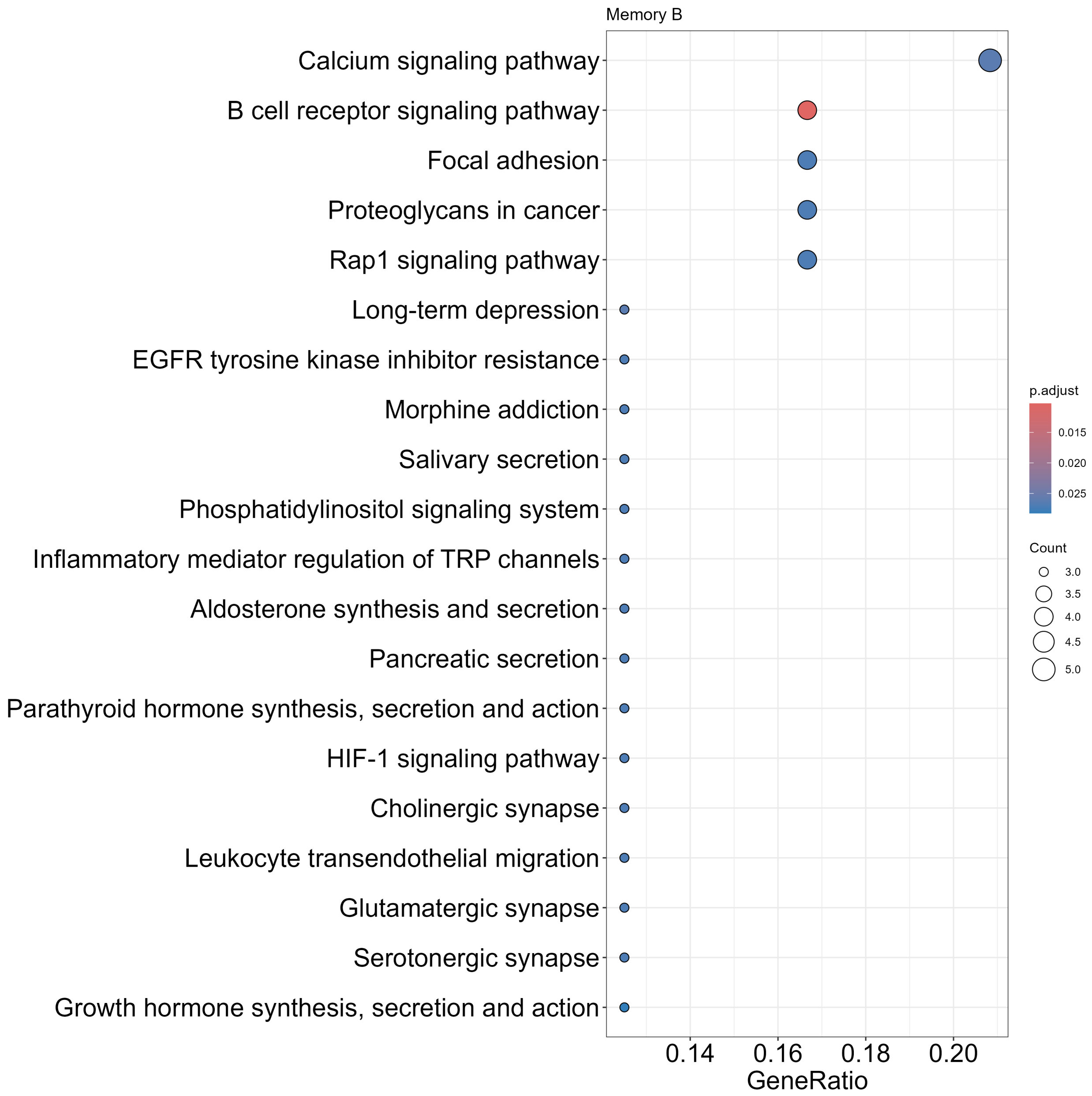

Supplementary Figure 16. Enriched KEGG Pathways in Memory B cells. Genes were ranked using node degrees from a TIGER-estimated Gene Regulatory Network (GRN). Over representation analysis on top 50 genes in conjunction with the KEGG pathway database, was utilized to identify pathways with significant enrichment. Related to Figure 4.

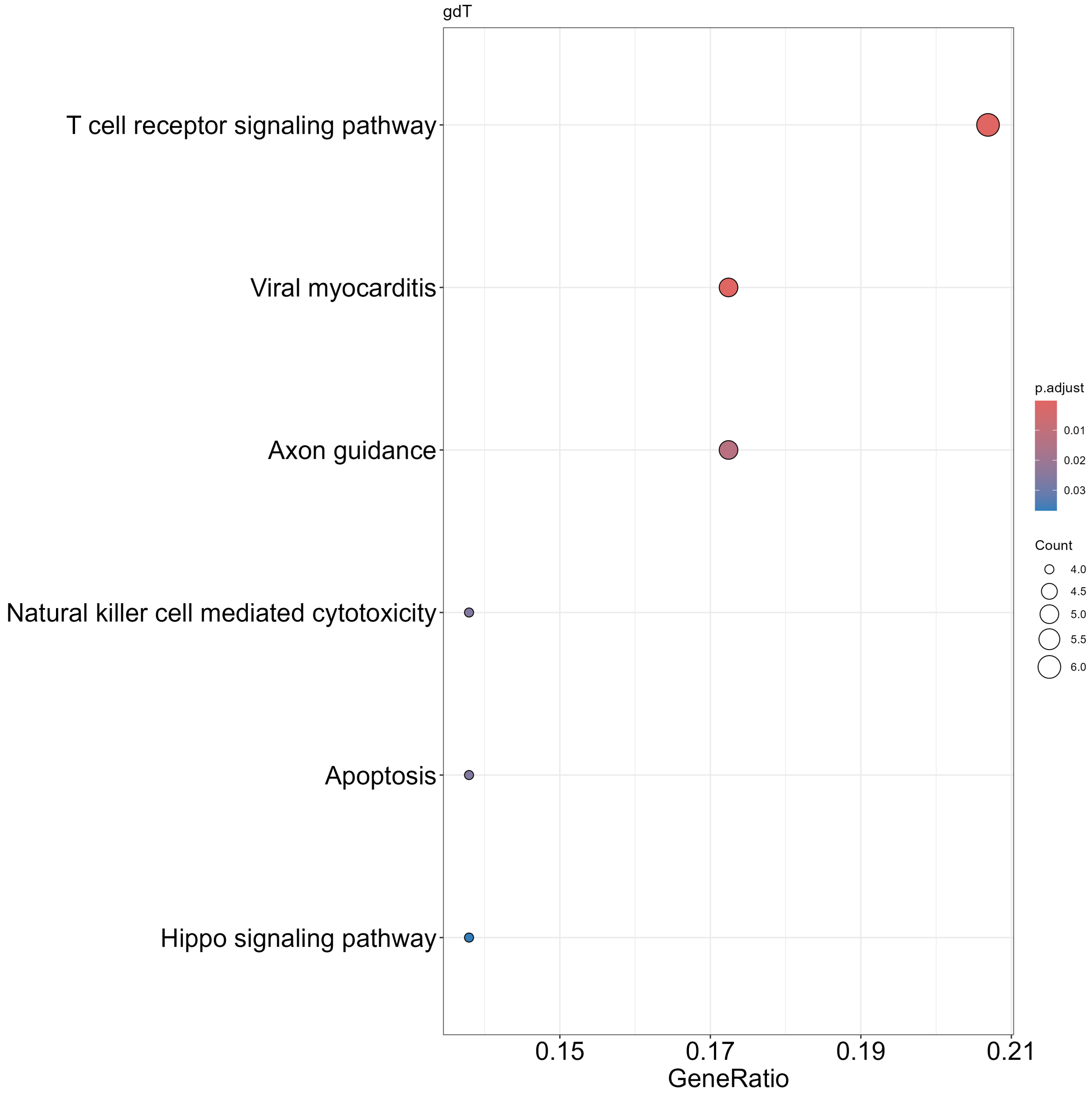

Supplementary Figure 17. Enriched KEGG Pathways in gdT cells. Genes were ranked using node degrees from a TIGER-estimated Gene Regulatory Network (GRN). Over representation analysis on top 50 genes in conjunction with the KEGG pathway database, was utilized to identify pathways with significant enrichment. Related to Figure 4.

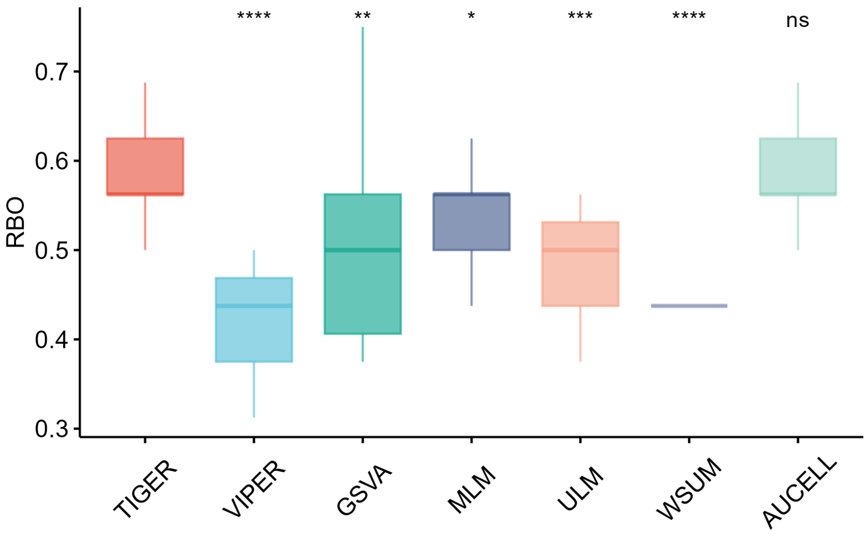

Supplementary Figure 18. TIGER outperforms other methods on ENCODE multi-omics mouse datasets (RNA-seq + DNase-seq). Related to Figure 4.

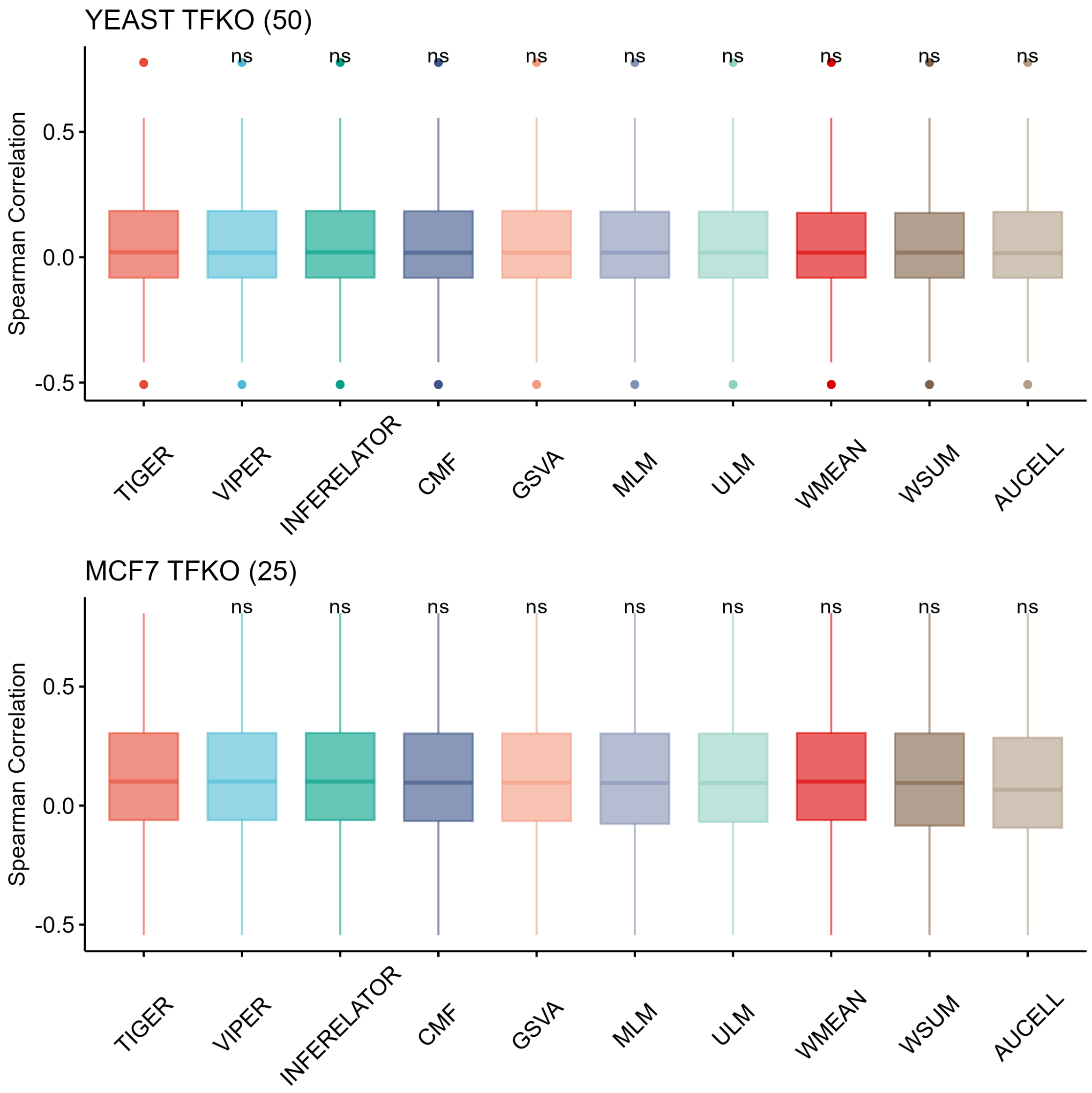

Supplementary Figure 19. Spearman Correlation Analysis of Transcription Factor Activity (TFA) Estimates and Gene Expression Levels. The correlation scores predominantly hover around zero across various datasets, such as Yeast and MCF7. Additionally, the Spearman correlation coefficients do not exhibit substantial variation among different TFA estimation methods.

2 Supplementary Table 1

Cell type	Top5 TFs	Literature Evidence
CD14 Mono	GLYR1/NR2C2/RFX2/HMGN3/ZNF644	
CD16 Mono	ZNF197/BRF2/ZBTB11/GLYR1/HMGN3	
CD4 Naive	MYC/ESR1/TCF7/STAT5B/ETS1	MYC: https://elifesciences.org/articles/53725<br>TCF7: https://rupress.org/jem/article-pdf/214/1/39/1134192/jem_20161046.pdf<br>STAT5B: https://www.ncbi.nlm.nih.gov/pmc/articles/PMC5247784/<br>ETS1:https://www.jimmunol.org/content/178/1_Supplement/S132.1
CD4 TCM	MYC/ZNF83/TCF7/MAF/MAZ	MYC:https://www.sciencedirect.com/science/article/pii/S258900422100729X
CD4 TEM	MYC/NME2/ETS1/THAP11/NFKB1	MYC:https://www.sciencedirect.com/science/article/pii/S258900422100729X
CD8 Naive	ZNF584/MYC/BACH2/TCF7/ZNF766	MYC:https://www.sciencedirect.com/science/article/pii/S1074761311005152?via%3Dihub<br>BACH2: https://www.nature.com/articles/ni.3441<br>TCF7:https://pubmed.ncbi.nlm.nih.gov/16424171/
CD8 TEM_1	ZNF83/TBX21/GATA3/ETS1/IRF3	ZNF83, TBX21, GATA3, ETS1: https://www.ncbi.nlm.nih.gov/pmc/articles/PMC5508124/
CD8 TEM_2	TBX21/BATF/STAT4/MAZ/GATA3	TBX21, BATF, GATA3: https://www.ncbi.nlm.nih.gov/pmc/articles/PMC5508124/<br>STAT4: https://www.jimmunol.org/content/177/11/7618.short
cDC	E2F4/HMGN3/ZNF165/STAT2/NR1I2	STAT2: https://www.jimmunol.org/content/197/1/326<br>HMGN3: https://www.nature.com/articles/ni.2516
gdT	TBX21/PRDM1/GATA6/SP4/STAT4	TBX21: https://www.frontiersin.org/articles/10.3389/fimmu.2020.00042/full<br>PRDM1(Blimp-1): https://www.jimmunol.org/content/199/7/2366<br>STAT4: https://onlinelibrary.wiley.com/doi/full/10.1111/imr.12918
HSPC	CBX2/TCF4/BRF2/NR1I2/ZKSCAN1	CBX2: https://www.sciencedirect.com/science/article/pii/S221112472200331X?via%3Dihub<br>TCF4: https://www.ncbi.nlm.nih.gov/pmc/articles/PMC6595260<br>ZKSCAN1: https://www.nature.com/articles/nature22370
Intermediate B	RFX5/ZNF318/ZNF165/ZNF92/IRF4	RFX5: https://www.sciencedirect.com/science/article/pii/S1074761300804677<br>ZNF318: https://www.pnas.org/doi/full/10.1073/pnas.1402739111
MAIT	MYC/NME2/TBX21/ETS1/GATA3	MYC: https://www.biorxiv.org/content/10.1101/2022.01.17.476571v1<br>TBX21: https://www.ncbi.nlm.nih.gov/pmc/articles/PMC8183572/#B63<br>GATA3: https://onlinelibrary.wiley.com/doi/10.1111/imcb.12281
Memory B	ADNP/PAX5/MEF2A/POU2F2/BACH2	PAX5: https://www.nature.com/articles/ni.2641<br>POU2F2 (OCT2): https://www.ncbi.nlm.nih.gov/pmc/articles/PMC4833274/<br>BACH2: https://www.nature.com/articles/ni.3493
Naive B	RFX5/ZNF318/SPIB/PAX5/LYL1	ZNF318: https://www.pnas.org/doi/full/10.1073/pnas.1402739111<br>SPIB: https://www.ncbi.nlm.nih.gov/pmc/articles/PMC2518887<br>PAX5: https://www.nature.com/articles/ni.2641<br>LYL1: https://pubmed.ncbi.nlm.nih.gov/16514064/
NK	HMGN3/TERF1/E2F1/TBX21/ETS1	E2F1: https://www.jimmunol.org/content/193/2/950<br>TBX21: https://www.sciencedirect.com/science/article/pii/S1074761304000767<br>ETS1: https://pubmed.ncbi.nlm.nih.gov/32350509/
pDC	RFX5/NR2C2/NME2/STAT2/HMGN3	RFX5: https://www.sciencedirect.com/science/article/pii/S1074761300804677?via%3Dihub<br>STAT2: https://www.jimmunol.org/content/197/1/326
Plasma	CBX2/IRF4/GATA6/NCOA3/NME2	IRF4: https://pubmed.ncbi.nlm.nih.gov/16767092/
Treg	ETS1/MAF/GATA3/BATF/ZEB1	ETS1: https://pubmed.ncbi.nlm.nih.gov/20855499/<br>MAF: https://www.nature.com/articles/s41590-019-0316-2<br>GATA3: https://www.ncbi.nlm.nih.gov/pmc/articles/PMC3204837/<br>BATF: https://pubmed.ncbi.nlm.nih.gov/28778586/

3 Supplementary Methods

3.1 The TIGER Model

TIGER uses Bayesian matrix factorization to estimate regulatory network and TFA levels. Generally, TIGER can be formulated as $\boldsymbol{X} = \boldsymbol{W}\boldsymbol{Z} + \boldsymbol{\epsilon}$, where $\boldsymbol{X}$ is the gene by sample ($M * N$) expression matrix, $\boldsymbol{W}$ is the gene by TF ($M * L$) regulatory network, $\boldsymbol{Z}$ is the TF by sample ($L * N$) activity matrix, and $\boldsymbol{\epsilon}$ is the random noise. TIGER assumes the gene expression $\boldsymbol{X}$ is a linear combination of inferred transcription factor activities specified by $\boldsymbol{Z}$ and regulation strengths specified by $\boldsymbol{W}$.

3.2 Identifiability Issue

If we do not add any constraints, the above model is non-identifiable. For example, parameters are said to be invariant under rotation when one can find an orthogonal rotation matrix $\boldsymbol{R}$, such that $\boldsymbol{W}\boldsymbol{Z} = (\boldsymbol{W}\boldsymbol{R})(\boldsymbol{R^T}\boldsymbol{Z}) = \boldsymbol{W}^*\boldsymbol{Z}^*$, where $\boldsymbol{R}\boldsymbol{R^T} = \boldsymbol{I}$. Also, there might be label switching (i.e., column permutations) and sign-flipping issues if we do not constrain the connection pattern and signs in $\boldsymbol{W}$. This will generate $L! * 2L$ equivalent solutions. [Anderson and Rubin, 1956], and [Liao et al., 2003] suggested that model identification can be achieved by imposing at least L^2 restrictions. We summarized these criteria as follows,

1. $\boldsymbol{W}$ must have full column rank.
2. Each column of $\boldsymbol{W}$ must have at least $L - 1$ zeros.
3. $\boldsymbol{Z}$ must have full row rank.
4. Constrain one parameter in each row of $\boldsymbol{Z}$ to take only positive or negative values.

We can check that the first two criteria add $L + L * (L - 1) = L^2$ constraints. The third criterion guarantees that each TF is unique so that the labels cannot be switched. Jointly, the first three conditions reduce the number of possible solutions from infinitely many to $2L$ produced by sign flipping. The fourth condition further reduces that number to 1 so that the resulting parameter space is identifiable up to a constant. (Note that we can always do $(\boldsymbol{W} * c)(1/c * \boldsymbol{Z})$, where c is some non-zero constant). The first three conditions are called NCA criteria [Liao et al., 2003], and proofs can be found in their supplementary notes.

3.3 Model Constraints

To solve the identifiability issue and achieve a biological meaningful solution, we can constrain the model by prior biological knowledge. This knowledge could be context-specific ChIP-seq data or literature-curated TF binding information, which imposes a set of zero restrictions on matrix $\boldsymbol{W}$. Very interestingly, this prior binding information automatically satisfies the identifiability criteria 1&2 because the gene regulatory network is known to be sparse and each TF regulates a specific set of genes. In other words, we already have a network $\boldsymbol{W}_0$ which is much sparser than the requirement of criterion 2, and it is full column rank (i.e., criterion 1). Criterion 3 implies the necessary condition that L (the number of TFs) must be less than N (the number of samples). If L is indeed less than M, the matrix $\boldsymbol{Z}$ is likely to have a full row rank for real biological data. Criterion 4 is also easy for TIGER because TIGER tries to differentiate activation and suppression events, which means it has to fix every element in $\boldsymbol{Z}$ to be strictly non-negative. In other words, we constrain the lowest TF activity level to zero. After all four criteria are satisfied, we still need to solve the rescaling problem. We followed the idea of [Sabatti and James, 2006] to define two new quantities, the average effect of each TF on the genes it regulates (regulon expression), and the average control strength over all experiments,

$$W^*_{ij} = \frac{\sum_n Z_{jn}}{N} * W_{ij}, \quad Z^*_{jn} = \frac{\sum_i |W_{ij}|}{\sum_i 1(W_{ij} \neq 0)} * Z_{jn}. \tag{1}$$

To obtain the point estimator of $\boldsymbol{W}$ and $\boldsymbol{Z}$, we calculate the posterior mean and subsequently apply the rescaling function 1 to compute the final values of $\boldsymbol{W}$ and $\boldsymbol{Z}$.

3.4 Bayesian Framework

TIGER uses the Bayesian framework to integrate prior knowledge with expression data. In this section, we will discuss the prior distribution choice and model fitting issues.

3.4.1 Prior distributions

The matrix $\boldsymbol{W}$ has been constrained to be sparse, so we only need to choose a prior for the non-zero elements. Note that this also implies the TIGER network is a subnetwork of the prior network. This is motivated by the fact that prior information from motifs, ChIP-seq data, or databases like DoRothEA, tend to be a superset of all possible regulatory events. To continue to refine the prior and enhance only those regulatory interactions with support in the data, we choose a sparse prior for $\boldsymbol{W}$ to shrink the context irrelevant edges to zero. Sparse priors are usually mixture models, and there are many options. A discrete mixture, such as spike-and-slab is hard to implement because of the combinatorics. TIGER uses a continuous mixture, which is easier to implement:

$$\begin{cases} W_{ij} \sim \mathcal{N}^{+}(0, \alpha_{ij}), \alpha_{ij} \sim \mathcal{IG}(a_\alpha, b_\alpha), & \text{if } W_{ij}^0 = +1 \\ W_{ij} \sim \mathcal{N}^{-}(0, \alpha_{ij}), \alpha_{ij} \sim \mathcal{IG}(a_\alpha, b_\alpha), & \text{if } W_{ij}^0 = -1 \\ W_{ij} = 0, & \text{if } W_{ij}^0 = 0 \end{cases} \tag{2}$$

where W_{ij}^0 is the prior knowledge encoding the regulatory event between TF i and target gene j, which is either positive (+1), negative (-1) or no regulation (0). $\mathcal{N}^{+}$ denotes the right-half normal distribution and $\mathcal{N}^{-}$ denotes the left half normal distribution. $\mathcal{IG}$ denotes the inverse-gamma function. If prior knowledge of edge sign is not available (i.e., $W_{ij}^0 = 1$ or 0), the sign constraint on the normal distribution will be removed.

Here, we assume each edge W_{ij} is independent of the others. The TF activities $\boldsymbol{Z}_n$ in each sample n jointly follow a multivariate normal distribution with a diagonal covariance matrix, implying each TF is independent of the others.This assumption simplifies the computation, but if protein (TF) interaction needs to be considered, we can generalize the covariance matrix $\boldsymbol{\Sigma_Z}$ by adding TF pairs to the off-diagonal part.

By default, TIGER uses $a_\alpha = b_\alpha = 1$, $a_\sigma = b_\sigma = 1$, and $\sigma_Z^2 = 100$. These default hyperparameters are appropriate for yeast, cancer cell line bulk RNA-seq, and PBMC scRNA-seq data. Other continuous mixtures such as the horseshoe prior [Piironen and Vehtari, 2017] can be used, but they induce a more complex posterior geometry and do not improve the accuracy of TF activity estimation. See section 3.4.3 for more discussion.

3.4.2 Likelihood

TIGER requires the matrix $\boldsymbol{X}$ to be a log-transformed normalized gene expression matrix. So the conditional likelihood function can be written as,

$$\mathcal{L} = \prod_n P(\boldsymbol{X}_n | \boldsymbol{W}, \boldsymbol{Z}_n, \sigma_\epsilon^2) = \prod_n \mathcal{N}(\boldsymbol{W}\boldsymbol{Z}_n, \sigma_\epsilon^2 \boldsymbol{I}) \tag{3}$$

3.4.3 Parameter estimation

To find the posterior distribution of $\boldsymbol{W}$ and $\boldsymbol{Z}$, we need to use numerical methods. MCMC is the most accurate, but we have a very large system with thousands of genes, samples (cells), and TFs. Thus, to achieve scalability to large datasets, we chose Variational Bayes [Wainwright et al., 2008] as TIGER's default numerical solver. Variational Inference (VI) approximates the posterior distribution using simple variational family distributions. Here, we use the mean-field Gaussian family, which assumes all the parameters have independent Gaussian distributions. The limitation of this approach is that it ignores the off-diagonal terms of the covariance matrix, which will lead to a systemic underestimation of marginal variances. However, the main aim of TIGER is to estimate TF activities (e.g., point estimators)and mean-field VI has been shown to successfully approximate the posterior mean [Blei et al., 2017].

Hierarchical priors (e.g, a scale mixture) can cause difficult posterior geometries (e.g., Neal's Funnel) in the limit when variance parameters approach zero [Neal, 2003]. Thus, in practice, we used non-centered reparameterization [Papaspiliopoulos et al., 2007] to replace equation 2 with $W_{ij} = \beta_{ij}\alpha_{ij}$ where

$$\begin{cases} \beta_{ij} \sim \mathcal{N}^{+}(0, 1), \alpha_{ij} \sim \mathcal{IG}(1, 1), & \text{if } W_{ij}^0 = +1 \\ \beta_{ij} \sim \mathcal{N}^{-}(0, 1), \alpha_{ij} \sim \mathcal{IG}(1, 1), & \text{if } W_{ij}^0 = -1 \\ \beta_{ij} = 0, \alpha_{ij} = 0, & \text{if } W_{ij}^0 = 0 \end{cases} \tag{4}$$

Another potential problem with the hierarchical model is multi-modality. This issue can be detected by inconsistent results of multiple runs. We did not observe this issue using our current prior setting. However, if we use a horseshoe prior the problem will emerge because horseshoe prior uses heavy-tailed half-Cauchy distribution to model the standard deviation. To alleviate the problem, the current best solution is to use a method called Bayesian stacking [Yao et al., 2018]. The main idea is to hit as many modes or separated regions as possible and then combine them using a weighted average of distributions. This method is computationally heavy, and our tests did not show any advantage of

applying the horseshoe prior on regulatory network inference or TF activity estimation. For these reasons, we do not recommend using a horseshoe prior.

3.4.4 Model checking

Checking a Bayesian model is usually not an easy task. Interestingly, [Vehtari et al., 2017] proposed an efficient and accurate method based on Importance Sampling to approximate leave-one-out cross-validation, named "Pareto Smoothed Importance Sampling (PSIS-LOO)". Using this method, we can quickly diagnose if there is any modeling fitting issue. Unfortunately, the conditional likelihood method cannot fit into this PSIS-LOO framework very well because the number of latent variables (i.e., TF activities) increases with the sample size. Thus, we recommend people split their own cross-validation sets if time permits.

3.4.5 Software

The analysis discussed above can be performed on the probabilistic programming platform STAN [Carpenter et al., 2017]. We implemented model inference using the R package "cmdStanR" version 0.5.3, and model checking using the R package "loo" version 2.5.1. "cmdStanR" uses the Automatic Differentiation Variational Inference (ADVI) [Kucukelbir et al., 2015] algorithm to minimize the KL divergence (i.e., maximize the evidence lower bound (ELBO)) between the proposal distribution and the posterior distribution in the real-coordinate space. The posterior mean is used to summarize the results.

3.5 Sign constraints revisit

In this section, we want to provide more details about the sign constraints in TIGER. TIGER's success comes from correctly differentiating positive and negative edge signs. Prior knowledge (e.g. from databases like DoRothEA) can provide edge sign information but it is not accurate. To best utilize the prior knowledge, TIGER incorporates the prior edge sign into the model, which means some of the edges in equation 2 are constrained as half-Normal distribution. However, it is important to decide which edge signs need to be constrained and which do not, because if we wrongly constrain them, TIGER's performance will decrease. Our strategy is to compute the partial correlation between the TF and gene based on their mRNA expression levels. If the partial correlation is consistent with DoRothEA, we will include the constraint on $\boldsymbol{W}$, otherwise, no constraint will be added, meaning TIGER will learn a new edge sign for us. Partial correlation was calculated using "GeneNet" R package version 1.2.16.

Another problem is after adding sign constraints, $\boldsymbol{X}_n = \boldsymbol{W}\boldsymbol{Z}_n + \boldsymbol{\epsilon}$ may not have a solution if gene expression $\boldsymbol{X}$ contains negative values. This is because most TFs are activators of gene expression and are not mathematically compatible with formulating gene expression in terms of negative values. However, negative expression value is very common after the log-transformation of tiny raw data or gene-wise standardization. We want to make TIGER a general tool that fits all types of RNA-seq, microarray, or even L1000 data; thus, we require the input of TIGER a normalized expression matrix, but people can choose their own appropriate pre-processing methods. Here, for the possible negative values after pre-processing, we propose two possible solutions. One is to add a small constant to the gene expression matrix so that it is fully non-negative. The other is to add a baseline expression value for every gene. Then the model becomes $\boldsymbol{X}_n = \boldsymbol{\mu} + \boldsymbol{W}\boldsymbol{Z}_n + \boldsymbol{\epsilon}$, where $\boldsymbol{\mu}$ is a vector of length M (number of genes). This transforms the problem into a typical factor analysis model. The second approach adds M more parameters to the posterior searching space. We have implemented the second approach as the default in TIGER, but we recommend the first approach if the user wants to accelerate the computation for large datasets.

The last problem is that the sign constraints make model fitting more difficult. ADVI will first log-transform constrained parameters to a non-constrained space. If the TFA or edge weights are zero, then the log transformation will create extremely small values, making it very hard to find an appropriate step size to search the parameter space. However, these are pathological cases that are infrequent in most use cases, and in general, do not disrupt model fitting for TIGER.

Supplementary References

[Anderson and Rubin, 1956] Anderson, T. and Rubin, H. (1956). Statistical inference in. In *Proceedings of the Third Berkeley Symposium on Mathematical Statistics and Probability: Held at the Statistical Laboratory, University of California, December, 1954, July and August, 1955*, volume 1, page 111. Univ of California Press.

[Blei et al., 2017] Blei, D. M., Kucukelbir, A., and McAuliffe, J. D. (2017). Variational inference: A review for statisticians. *Journal of the American statistical Association*, 112(518):859–877.

[Carpenter et al., 2017] Carpenter, B., Gelman, A., Hoffman, M. D., Lee, D., Goodrich, B., Betancourt, M., Brubaker, M., Guo, J., Li, P., and Riddell, A. (2017). Stan: A probabilistic programming language. *Journal of statistical software*, 76(1).

[Kucukelbir et al., 2015] Kucukelbir, A., Ranganath, R., Gelman, A., and Blei, D. (2015). Automatic variational inference in stan. *Advances in neural information processing systems*, 28.

[Liao et al., 2003] Liao, J. C., Boscolo, R., Yang, Y.-L., Tran, L. M., Sabatti, C., and Roychowdhury, V. P. (2003). Network component analysis: reconstruction of regulatory signals in biological systems. *Proceedings of the National Academy of Sciences*, 100(26):15522–15527.

[Neal, 2003] Neal, R. M. (2003). Slice sampling. *The annals of statistics*, 31(3):705–767.

[Papaspiliopoulos et al., 2007] Papaspiliopoulos, O., Roberts, G. O., and Sköld, M. (2007). A general framework for the parametrization of hierarchical models. *Statistical Science*, pages 59–73.

[Piironen and Vehtari, 2017] Piironen, J. and Vehtari, A. (2017). Sparsity information and regularization in the horseshoe and other shrinkage priors. *Electronic Journal of Statistics*, 11(2):5018–5051.

[Sabatti and James, 2006] Sabatti, C. and James, G. M. (2006). Bayesian sparse hidden components analysis for transcription regulation networks. *Bioinformatics*, 22(6):739–746.

[Vehtari et al., 2017] Vehtari, A., Gelman, A., and Gabry, J. (2017). Practical bayesian model evaluation using leave-one-out cross-validation and waic. *Statistics and computing*, 27(5):1413–1432.

[Wainwright et al., 2008] Wainwright, M. J., Jordan, M. I., et al. (2008). Graphical models, exponential families, and variational inference. *Foundations and Trends® in Machine Learning*, 1(1–2):1–305.

[Yao et al., 2018] Yao, Y., Vehtari, A., Simpson, D., and Gelman, A. (2018). Using stacking to average bayesian predictive distributions (with discussion). *Bayesian Analysis*, 13(3):917–1007.
